# Depolymerisation of *γ*‐Valerolactone Organosolv Lignins with Unsupported Molybdenum‐Based Catalysts

**DOI:** 10.1002/cssc.202500643

**Published:** 2026-02-08

**Authors:** Silja Känsäkoski, Saravanan Kasipandi, Taina Ohra‐aho, Tom Wirtanen, Juha Lehtonen, David Martin Alonso, Francisco Vila, Sari Rautiainen

**Affiliations:** ^1^ VTT Technical Research Centre of Finland Ltd. Espoo Finland; ^2^ EQS Group (Sustainable Energy and Chemistry Group) Institute of Catalysis and Petrochemistry (CSIC) Madrid Spain

**Keywords:** depolymerisation, heterogenous catalysis, hydrogenolysis, lignin, molybdenum

## Abstract

Lignin is an attractive feedstock for a wide variety of applications ranging from aromatic chemicals and transportation fuels to resins and coatings. Emerging biorefinery concepts, like the organosolv process, enable the separation of all the lignocellulose components, and moreover, produce lignins of high quality and purity susceptible to valorisation by depolymerisation. In this work, we focus on the depolymerisation of lignins obtained by *γ*‐valerolactone (GVL) organosolv fractionation of four biomass feedstocks, eucalyptus, white birch, sugarcane bagasse and Scots pine. We demonstrate that lignins extracted with the GVL process are depolymerised using unsupported molybdenum‐based catalysts under reductive conditions in supercritical ethanol. As a result, over 90% yields of low‐molecular‐weight lignin oils are obtained with minimal char formation, yields of the aromatic monomers being 7–16 wt%. Furthermore, the design of experiments method is used to analyse the effect of depolymerisation conditions, catalyst, hydrogen loading and temperature, on the yields and properties of the product fractions. Notably, we show that the properties of the lignin oils and monoaromatics can be tuned towards the targeted application by modifying the depolymerisation conditions.

## Graphical abstract

Lignins from *γ*‐valerolactone organosolv fractionation were depolymerised in ethanol using unsupported molybdenum catalysts. Over 90% yields of low‐molecular‐weight lignin oil were obtained with minimal char formation, yields of the aromatic monomers being 7–16 wt%. Furthermore, we show how the product properties, like molecular weight and hydroxyl content, can be tuned by modifying the depolymerisation conditions.

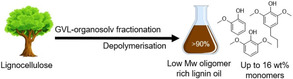

## 1 Introduction

New renewable alternatives for fuels, chemicals and materials are required to decrease the stress and reliance society has on fossil resources. Biomass, lignocellulosic biomass in particular, is an attractive and accessible renewable carbon feedstock for value‐added products like chemicals and fuels [1, 2, 3]. Lignocellulosic biomass is made up of cellulose (35%–50%), hemicellulose (25%–30%), and lignin (15%–30%) [4]. Of these, lignin is the only one with an aromatic skeleton, making it a noteworthy and underutilised source for aromatic chemicals and materials [5]. Although lignin is an attractive feedstock, its depolymerisation into monomers has challenged researchers for decades due to its complex and heterogeneous structure in addition to its tendency to condensate to new oligomeric and polymeric structures upon processing [6].

**FIGURE 1:**
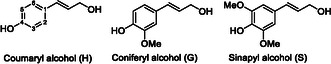
Monolignol structures.

**FIGURE 2:**
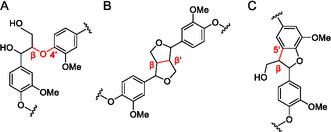
Interunit linkages, (A) *β*‐O‐4, (B) *β*‐*β*, (C) *β*‐5.

Lignin is a complex natural polymer that is mainly formed of three phenolic monomers: *p*‐coumaryl alcohol (H‐unit), coniferyl alcohol (G‐unit) and sinapyl alcohol (S‐unit) (Figure 1). The relative amount of these monolignols varies greatly depending on the biomass source. The lignin biomass sources can be divided into three categories: softwood, hardwood, and herbaceous (e.g. agricultural crops) lignin [7]. Softwood lignin is mainly comprised of G units, whereas hardwood lignin is composed of G and S units. Herbaceous lignin contains all H, G and S units [8]. The monolignols are connected to each other through various interunit linkages, some of the most abundant being *β*‐O‐4, *α*‐O‐4, *β*‐*β*, 4‐O‐5, *β*‐1 and 5‐5 (Figure 2). In general, C—C bonds in the interunit linkages are harder to break compared to C—O bonds. Lignin feedstocks containing low C—C bond content and high amounts of *β*‐O‐4 linkages are considered ideal for lignin depolymerisation [9]. Therefore, the structure of the lignin has a crucial role in the outcome of the depolymerisation.

In conventional pulping processes, like Kraft, sulphite and soda pulping, lignin is often seen as a hindrance for the valorisation of cellulose, and consequently, the goal of these processes is the removal of lignin from biomass to create a processable cellulose fraction with no consideration for the properties of the lignin [10]. Of these fractionation methods, Kraft pulping is the dominant technique, accounting for approximately 80% of chemical pulping processes [11]. Considerable degradation of lignin takes place during the harsh Kraft pulping process, resulting in low content of *β*‐O‐4 linkages and a high degree of condensation, making Kraft lignin a poor candidate for further depolymerisation into monoaromatic chemicals. Furthermore, the lignin is present in black liquor in the Kraft process, being part of the chemical circulation of a pulp mill and to obtain pure Kraft lignin, a dedicated separation process, such a LignoBoost, is required [9, 12].

Alternative fractionation processes have been developed to enable valorisation of all the lignocellulose fractions and to account for the poor‐quality lignin. A promising process is the organosolv process, in which lignocellulose is treated in an organic solvent, causing the lignin and hemicellulose to be solubilised [9]. Many solvents can be used for this, including methanol, ethanol, glycerol, organic acids, ketones, ethers and esters (e.g. *γ*‐valerolactone) [13]. The extraction using an organic solvent primarily breaks *α*‐aryl ether bonds, whereas lower amounts of *β*‐aryl ether linkages are cleaved, resulting in a lignin rich in *β*‐O‐4 bonds [14]. A major advantage of the organosolv pretreatment method is that it enables further valorisation of all lignocellulosic components as it forms three product streams: cellulose‐rich pulp, solubilised hemicellulose and precipitated lignin [9].

Of the many solvents that can be used in organosolv pretreatment, *γ*‐valerolactone (GVL) has shown promise as a renewable solvent with high lignin solubility, producing high‐quality lignin abundant in *β*‐ether linkages [15]. GVL is a non‐toxic, green, and non‐volatile solvent that is stable at ambient conditions, making it safe for large‐scale storing and transportation [16]. Fang and Sixta showed fractionation of lignocellulose using GVL/water mixtures; about 40% of the lignin was recovered using microwave heating for 2 h at 170°C [17]. The addition of sulfuric acid decreased the temperature and time needed; however, the lignin yield was not markedly improved. Dumesic's group developed a process concept for GVL fractionation enabling high biomass loading (20 wt%); over 70% of lignin was isolated after fractionation with 0.1 M sulfuric acid at 125°C during 3 h [18]. The fractionation process has been successfully applied for various biomass sources, including eucalyptus, maple, poplar, corn, and bamboo [19, 20, 21, 22]. The cellulose fraction can be used as feedstock for glucose‐derived chemicals or dissolving pulp, and the hemicellulose can be converted into furfural, a key biomass‐derived building block [18]. The valorisation of the lignin fraction would provide a base case for a biorefinery concept where all three main fractions of lignocellulosic biomass are converted into high‐value products.

Valorisation of lignin as monomeric or oligomeric products requires depolymerisation or fractionation of the lignin. Many different catalytic techniques have been employed in the depolymerisation of lignin into lower molecular weight products, such as oxidative, reductive, and acid catalysed processes [23]. Reductive catalytic depolymerisation (RCD) is considered a highly promising method because the presence of hydrogen aids in controlling the recondensation of reactive intermediate species by saturating reactive double bonds. The source of hydrogen can be gaseous H_2_, or a hydrogen donating solvent (isopropanol, methanol, ethanol, formic acid) can also provide active hydrogen species [24]. Low‐carbon‐number alcohols such as ethanol, isopropanol, and n‐butanol can be produced from the fermentation of lignocellulosic wastes, which could improve the sustainability and profitability of biorefineries [25]. Using both hydrogen‐donating solvents and H_2_ for lignin depolymerisation can reduce char formation and give high monomer yields [25]. Supercritical ethanol has been found to produce high monomer yields with no char formation, making it an attractive solvent for producing aromatics or monophenols [26].

Catalysts are often employed to improve lignin oil and monomer yields and to decrease char formation. Non‐precious metal catalysts are usually cheaper options than precious metal catalysts, hence more feasible to scale up for industrial applications. Molybdenum‐based catalysts are attractive for aromatics production as they have been found to cleave the ether bond rather than hydrogenate aromatic rings [27]. Ma et al. studied the depolymerisation of Kraft lignin in supercritical ethanol over supported Mo‐based catalysts at 280°C [28, 29]. In a typical experiment, 1 g of kraft lignin, 0.5 g of catalyst and 100 mL ethanol were loaded into the reactor, which was pressurised to the desired pressure with H_2_ and heated to 280°C, holding this temperature for 6 h. The molybdenum carbide catalyst had the highest yields of 1.64 g/g of C6‐C10 chemicals, including esters, phenols, aliphatic alcohols, benzyl alcohols and arenes, part of which originated from the condensation of ethanol. Metallic molybdenum and molybdenum nitride showed great activity as well. Overall, molybdenum carbide catalysts show promise as a low‐cost substitute to noble metal catalysts thanks to the unique D‐band electron properties that resemble those of noble metals [30]. Molybdenum phosphides have not been examined as rigorously as molybdenum carbides. Chowdari et al. used a molybdenum phosphide on activated carbon in the hydrotreatment of Kraft lignin [31]. In their procedure, 15 g Kraft lignin and 0.75 g catalyst were loaded into the reactor, which was pressurised to 100 bar with H_2_ and heated to the desired temperature (400–500°C). This catalyst produced 61.2% of lignin oil and 38.7 wt% of monomers. Chen et al. synthesised phosphided Mo/sepiolite catalysts that were used to depolymerise Kraft lignin in ethanol [32]. Typically, 1 g Kraft lignin, 0.5 g catalyst and 40 mL ethanol were loaded into the reactor and heated to 290°C. The highest yield of phenolics was 33.4% which was produced using the 5P‐Mo/SEP catalyst.

A limited number of studies can be found on the catalytic depolymerisation of GVL lignins. Song et al. performed oxidative conversion of maple GVL lignin using 500 mg lignin and 200 mg of catalyst (Au nanoparticles supported on a basic Li–Al layered double hydroxide) in 10 mL dimethylformamide with oxygen flow at 120°C for 24 h [33]. Monomer yields of up to 40% were reached in these experiments. Karunasinghe et al. performed oxidative depolymerisation on maple wood GVL lignin, using a AuPd bimetallic catalyst supported on a Li‐Al layered double hydroxide support under oxidative conditions at 120°C [34]. For the experiments, 250 mg lignin, 100 mg catalyst and 15 mL dimethylformamide were added to a batch reactor. The best catalyst yielded 27% of aromatic monomers. McClelland et al. studied maple wood lignin extracted with GVL in supercritical methanol, using porous copper metal oxide catalyst [35]. In their typical set‐up, 100 mg catalyst, 100 mg lignin, and 2.4 g methanol were placed in a batch reactor and heated to 300°C with a reaction time of 15 min to 4 h. Lignin monomer yields of 20% were recovered at a 4 h reaction time. To our knowledge, only one previous study reports the hydrogenolysis of GVL lignins; the hydrogenolysis of corn stover GVL lignin was studied in a two‐step process using a Ru/C catalyst by Luterbacher et al. [36]. In the first step, the lignin was dissolved to form a mixture of lignin (10%), THF (80%), H_3_PO_4_ (8.5%) and H_2_O (1.5%), which was treated at 150°C under hydrogen. The THF was evaporated and replaced with heptane. This mixture was then treated in the second step at 250°C in the presence of Ru/C and H_2_. This two‐step depolymerisation process of GVL lignin produced up to 38 wt% of different types of monomers.

Lignin depolymerisation, regardless of the method applied, produces a complex mixture of products, the separation of which requires tedious multi‐step processes and results in low yields of the purified single product. Various strategies have been developed for the downstream processing of the product mixtures, such as chemocatalytic or biological funnelling of the mixture to reduce the complexity [37]. The characteristics and valorisation of the oligomeric fractions present in the lignin oils have received much less attention, even though these fractions could be upgraded in existing industrial processes or used in various applications like resins, polymers and adhesives [38]. Despite the growing understanding of the effect of the depolymerisation process on the monomeric products, the effect on the oligomeric products is far less established.

Herein, we present the isolation, characterisation and reductive depolymerisation of GVL lignins from various lignocellulosic feedstocks into low‐molecular‐weight lignin oils. The depolymerisation is carried out by ethanolysis and combined ethanolysis/hydrogenolysis in batch reactors using unsupported molybdenum carbides and phosphides as catalysts. To our knowledge, this is the first report showing the RCD of GVL lignins with non‐precious metal catalysts. We demonstrate that lignin extracted with GVL organosolv fractionation can be depolymerised into high yields of oligomeric lignin oils with minimal char formation using unsupported molybdenum‐based catalysts. Furthermore, based on a design of experiments study, we show that the properties of the lignin oils can be tuned by modifying the depolymerisation conditions to match the desired application.

## 2 Experimental Section

This section provides an overview of the materials and methods used. Please refer to the Supporting Information for detailed experimental descriptions.

### 2.1 Materials

Biomass samples were received from Stora Enso. Reagents were commercially purchased and used as such without further purification. Ethanol (Altia, ≥99.5%), tetrahydrofuran (VWR chemicals, ≥99% stabilised ACS), ammonium heptamolybdatet etrahydrate (NH_4_)_6_Mo_7_O_24_·4 H_2_O (VWR chemicals, 81.0–83.0%), diammonium hydrogen phosphate (NH_4_)_2_HPO_4_ (Merck, ≥99% ACS reagent grade), citric acid monohydrate (VWR chemicals, ≥99% ACS), 1‐cyanoguanidine (Sigma–Aldrich, ≥98.0%), 2,4,6‐triamino‐1,3,5‐triazine (Sigma–Aldrich, 98.5%–101.5%), cyanuric acid (Sigma–Aldrich, ≥98.0%), phenol (Sigma–Aldrich, 99%), o‐cresol (Sigma–Aldrich, 99.5%), m‐cresol (VWR chemicals, 99%), 4‐ethylphenol (Sigma–Aldrich, 99%), 4‐propylphenol (Aldrich Chemistry, 99%), 2‐propylphenol (Aldrich, 98%), guaiacol (Sigma–Aldrich, 99%), 4‐methylguaiacol (Sigma–Aldrich, 99%),4‐ethylguaiacol (SAFC, 98%), 4‐propylguaiacol (Sigma–Aldrich, 99%), eugenol, (Sigma–Aldrich, 99%), isoeugenol (Sigma–Aldrich, 98%), vanillin (Fluka AG, 99%), syringol (Thermo Fisher Scientific, 99%), 4‐methylsyringol (Sigma–Aldrich, 97%), N_2_ (Woikoski, 99.999%), H_2_ (Woikoski, 99.999%), 1‐butanol (Sigma–Aldrich, 99.8%). EtOH organosolv lignin was obtained from Fraunhofer, prepared by an ethanol‐water organosolv process.

### 2.2 Lignocellulose Fractionation

GVL lignin samples were produced from 4 different feedstocks, two hardwoods (white birch and eucalyptus globulus), one softwood (pinus sylvestris) and one agricultural residue sugarcane bagasse (SCB). In a typical fractionation experiment, biomass and liquid solution of GVL/water and 0.1 M sulfuric acid were added to a 1 L reactor and heated to the reaction temperature (125°C or 130°C) for 60–90 min. At the end of the reaction, the liquid was separated from the solid using vacuum filtration. The cellulose was analysed to determine the amount of hemicellulose and lignin extracted into the liquid phase. Cellulose yield is calculated based on the original weight of dry wood, while hemicellulose and lignin extraction yields are calculated based on their content in the feedstock. For the scale‐up reactions, we used a 20 L recirculation reactor built by EDIBON.

To precipitate the lignin from the liquid fraction, water was added to a ratio of water/GVL = 8, and the solution was centrifuged. The lignin was washed with hot water until the GVL and carbohydrate content were <0.5 wt% and air dried. After lignin precipitation, the aqueous solution was concentrated, and the C5 sugars were converted using a continuous flow reactor at 225°C to furfural and solid residue containing humins [39].

### 2.3 Lignin Analysis

The lignins were analysed for molecular weights, H/G/S‐ratio, interunit linkages and methoxy group content (Se). Size exclusion chromatography (SEC) in alkali solution was used to analyse the molecular weight of the lignin. For the analysis of lignin H/G/S‐ratio, thermochemolysis with tetramethyl ammonium hydroxide (TMAH) was performed. Amounts of aryl ether (*β*‐O‐4), phenyl coumaran (*β*‐5), and resinol (*β*‐*β*) type interunit linkages were quantified from the lignin samples using ^1^H‐^13^C HSQC NMR spectroscopy based on the literature assignments [40]. The methoxy group content was determined by a headspace GC equipped with an electron capture detector (HS‐GC/ECD) following the method by Baker with some modifications [41].

### 2.4 Catalyst Preparation

A total of five unsupported molybdenum‐based catalysts were prepared, three carbides and two phosphides. Two of the molybdenum carbides, MoC‐1 and MoC‐2, were prepared by following the method reported by Tang et al., using ammonium heptamolybdate, cyanuric acid and melamine as the precursors, followed by pyrolysis at 750°C and 650°C, respectively [42]. The third molybdenum carbide, MoC‐3, followed the preparation method reported by Ma et al. in which dicyanamide and ammonium heptamolybdate precursors were pyrolysed at 750°C [43].

For the first molybdenum phosphide, MoP‐1, ammonium heptamolybdate and diammonium hydrogen phosphate were used as precursors [44]. The second molybdenum phosphide catalyst, MoP‐2, was prepared by following the method reported by Cheng et al. [45], which was the same as the previous method with the addition of citric acid. Both catalysts were first calcined at 500°C followed by reduction in a fixed‐bed reactor under H_2_ flow at 650°C.

### 2.5 Catalyst Characterisation

Nitrogen physisorption using a Micromeritics 3Flex system was used to determine the brunauer‐emmett‐teller (BET) surface area of the catalysts. X‐ray diffraction (XRD) analysis was performed using PANalytical X’Pert Pro MPD with CuK*α* (1.5419 Å) radiation and focusing optics with programmable divergence and antiscatter slits set to 10 mm irradiated length. Scanning electron microscopy (SEM) was performed using a Carl Zeiss Merlin electron microscope to analyse the microscopic structure and morphology. Transmission electron microscopy (TEM) was done using ThermoFisher Scientific Talos F200X G2 STEM operated at 200 kV and equipped with the four SDD Super‐X energy dispersive spectrometer (EDS) detectors. The morphology and crystallinity of catalysts were investigated with S/TEM images and selected area electron diffraction.

### 2.6 Depolymerisation Experiments

The batch experiments were performed in a 200 mL Büchi high‐pressure autoclave reactor (Hastelloy) and in a 1 L Autoclave Engineers autoclave equipped with an inner tube (stainless steel 316). In a typical run, the reactor was charged with lignin (2–4 g), catalyst (0–20 wt%), and ethanol (80–160 mL). The air in the reactor was purged three times with nitrogen, then pressure tested. The reactor was purged another three times with hydrogen and pressurised to the desired pressure with hydrogen (0–40 bar H_2_) at ambient temperature. The mixture was heated to the required temperature (280°C, 315°C, 350°C). After reacting for 4 h with 600 RPM stirring, the reactor was cooled to room temperature. The reaction mixture was collected and filtered, and ethanol was evaporated from the filtrate, producing the ethanol‐soluble lignin oil containing monomers and low molecular weight oligomers. The remaining ethanol‐insoluble fraction after filtration was dissolved in THF, filtered and evaporated to isolate the residual lignin containing heavy molecular weight products. The remaining solid consisted of catalyst and char, and the bio‐char yield was calculated by subtracting catalyst mass from the total solids. Figure S7 in the Supporting Information displays the process flow for the sample treatment.

### 2.7 Product Analysis

Yields of the product fractions, lignin oil, residual lignin and char, were calculated according to Equation (1), relative to the initial lignin mass:

(1) $$\mathrm{product}\;\mathrm{yield}\;(\mathrm{wt}\%)=m_{\mathrm{produc}t}/m_{\mathrm{lignin}}\times 100$$

The monomer yields were measured directly from the ethanol filtrate using a gas chromatograph equipped with a mass selective detector. SEC was performed on both the lignin oil and residual lignin fractions to determine the molar weights. To determine the reduction in Mw during depolymerisation, the Mw of lignin feedstock before (Mw_feedstock_) and the Mw of the product lignin oil (Mw_lignin oil_) were used to calculate the change:

(2) $$\%\mathrm{reduction}\;\mathrm{in}\;\mathrm{Mw}=({\mathrm{Mw}}_{\mathrm{feedstock}}-{\mathrm{Mw}}_{\mathrm{lignin}\;\;\mathrm{oil}})/{\mathrm{Mw}}_{\mathrm{feedstock}}\times 100\%$$

The hydroxyl contents were determined from select samples using ^31^P NMR measurements with 2‐chloro‐4,4,5,5‐tetramethyl‐1,3,2‐dioxaphopholane as the phosphitylation reagent. The ESI provides more details on the ^31^P‐NMR analyses, and Table S9 displays the results and chemical shift ranges according to Granata et al. [46].

## 3 Results and Discussion

### 3.1 GVL Fractionation

Organosolv fractionation of eucalyptus, white birch, Scots pine and SCB was performed in 70/30 GVL/water as solvent. Sulfuric acid was used at 0.1 M concentration to facilitate the hydrolysis and solubilisation of hemicellulose while maintaining mild temperatures, 125°C–130°C, and short reaction times, 60–90 min. The reaction conditions were optimised to maximise the yield and purity of all three main fractions of lignocellulosic biomass (cellulose, hemicellulose, and lignin) while working at high biomass loading (20 wt%) to improve the economics of the process. The high biomass loading results in sulfuric acid use of less than 4 wt% of the biomass (39 kg of sulfuric acid per 1000 kg of biomass).

Prior to the fractionation, the feedstocks were analysed for their composition (Table S2). GVL extraction of eucalyptus resulted in >95% of the hemicellulose and >90% of the lignin present in the feedstock being extracted into the liquid phase. The remaining solid cellulose fraction amounted to 46.8 wt% of the biomass with >90% purity (Table 1, GVL Eucalyptus). Similar results were obtained for white birch; >92.5% of hemicellulose and >96.5% of lignin were extracted into the liquid phase, yielding 43.7 wt% as cellulose fraction with 92.9% purity (GVL White birch 1). In the case of SCB at 20 wt% biomass loading, 83.8% of the hemicellulose and 92% of the lignin were extracted, yielding 47.6 wt% of the feedstock as cellulose fraction with 82% purity (GVL SCB 1). Slightly higher lignin extraction and cellulose yields were obtained at 125°C in 90 min reaction time (SCB 2). For Scots pine, the lignin yield was considerably lower, only 48.6%, while hemicellulose extraction was similar to the other feedstocks, 87.6% (GVL Scots pine). The cellulose yield for pine was 57.7% with a purity of only 68.4%. Higher lignin extraction (81%) and cellulose purity (86%) could be achieved by increasing the reaction time to 120 min, GVL/water ratio to 80/20 and acid loading to 0.125 M; however, this caused the degradation of the hemicellulose sugars and the cellulose, increasing the unwanted formation of humins.

**TABLE 1:** Lignocellulose fractionation conditions and yields of different fractions.^a^

Feedstock	Temp, °C	Reaction time, min	Lignin extracted (wt% of theor.)	Klason lignin yield (wt% of theor.)	Cellulose yield (wt% of dry biomass)	Cellulose purity (wt% C6 sugars)	Hemicellulose yield (wt% of theor.)
GVL eucalyptus	130	60	90.8	84.8	46.8	90.1	95.0
GVL white birch 1	130	60	96.5	82.8	43.7	92.9	92.5
GVL white birch 2^b^	125	90	87.7	80.5	46.4	91.8	91.6
GVL SCB 1	130	60	92.0	81.5	47.6	82.0	83.8
GVL SCB 2	125	90	88.8	86.6	51.5	86.0	81.6
GVL Scots pine	130	60	48.6	48.5	57.7	68.4	87.6

a Reaction conditions: solvent GVL/water = 70/30, sulfuric acid concentration = 0.1 M, 20 wt% biomass loading. Lignin and hemicellulose yields are calculated based on their content in the given feedstock (Table S2), and cellulose yield is given as wt% of dry biomass.

b Performed in a 20 L reactor.

To precipitate the lignin from the solution, water was added to achieve a ratio of water/GVL = 8 [18]. After recovering the lignin by centrifugation, it was washed with hot water to recover the GVL retained by the lignin. Similar lignin yields (>80%) were obtained for the hardwoods and the SCB, while a lower yield was obtained for pine (48.6 wt%) because of the poor extraction achieved during the fractionation. In all cases, the purity of the lignin was high with less than 0.5 wt% of sugars present and less than 0.2 wt% of ashes (Table S3).

Valorisation of all the lignocellulose fractions is key to a feasible fractionation process. The high purity of the cellulose fractions, especially with the hardwoods white birch and eucalyptus, indicates that the pulp could be used as raw material to produce dissolving pulp. The viscosity of GVL white birch 1 pulp fraction was measured at 450 ml/g (CED viscosity SCAN‐CM 15:99). The promising result prompted us to further scale up the fractionation process of white birch in a 20 L reactor equipped with liquid recirculation. To reduce the degradation of the cellulose and improve its quality as feedstock for dissolving pulp, the fractionation temperature was reduced to 125°C, and the reaction time increased to 90 min (GVL White birch 2). This decreased the hemicellulose slightly (91.6%) and lignin extraction yields (87.7%) but increased the pulp viscosity to >700 ml/g.

**FIGURE 3:**
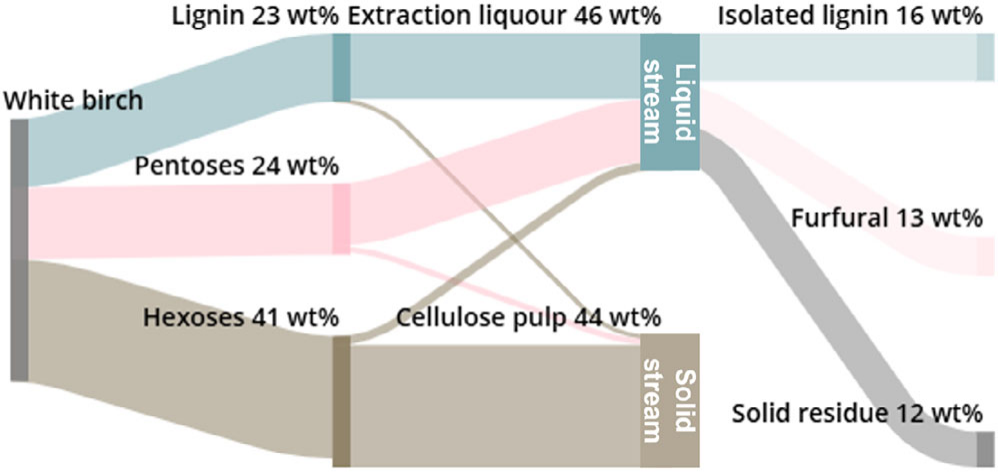
Mass balances in the GVL fractionation of white birch components into lignin, furfural and cellulose pulp.

In the case of SCB and pine, the degradation of the pulp led us to explore its valorisation as feedstock to produce levulinic acid, a key biomass‐derived chemical building block [18]. To minimise the use of additional solvents, the cellulose was converted into levulinic acid using the same solvent used for washing of the pulp, demonstrating the versatility of the GVL as a solvent. Up to 50 mol% levulinic acid was produced using sulfuric acid as an acid catalyst at 15 wt% biomass loading, and the yield increased to 70 mol% by reducing the solid concentration (see Supporting Information for details). Valorisation of the hemicellulose fraction was studied by the production of furfural directly from the aqueous hemicellulose solution after lignin precipitation. Using the sulfuric acid originating from the fractionation, the solution was rapidly heated to 225°C, resulting in over 75 mol% furfural yields based on the xylose content (Figure S6). In the case of white birch, which is rich in xylose, the yield corresponds to 13 wt% of white birch (Figure 3). As a side product, about 12 wt% solid residue containing humins and precipitated lignin was obtained. Detailed composition of the streams is given in Figure S5. These results indicate that the hemicellulose fraction can easily be valorised directly from the extraction liquor without additional catalyst or separation of the sugars. Figure 3 depicts the lignocellulose components, their separation and the overall product yields in the GVL fractionation of white birch.

### 3.2 Lignin Characterisation

Before starting to investigate the depolymerisation of the lignins obtained from the GVL fractionation, the lignins were characterised to determine the molecular weight, interunit linkages and monolignol content. A eucalyptus organosolv lignin (EtOH eucalyptus), obtained from the Fraunhofer pilot plant using an ethanol‐water organosolv fractionation with added sulfuric acid, was used as a reference and characterised along with the GVL lignins.

The lignin molecular weights ranged from 2100 up to 4600 Da (Table 2). The GVL SCB 2 lignin exhibited the lowest molecular weight, while the GVL Scots pine had the highest. The molecular weight of isolated lignins depends on wood species, extraction method and extraction condition intensity, meaning they can vary greatly [47]. According to literature, GVL lignin molecular weights commonly range between 1900 and 5000 Da [15, 48, 49]. Due to the SEC column being changed, all molecular weights in this work cannot be directly compared; however, the molecular weight of the starting lignin and its resulting lignin oil were always measured with the same column.

**TABLE 2:** Results of the lignin characterisation: Mw, H/G/S ratio, interunit linkages, and methoxy content of different lignins.^a^

Entry	Lignin	Mw, Daltons	Monolignol%			Interunit linkage (% from aromatic units)			OCH_3_, mmol/g
			H%	G%	S%	*β*‐O‐4	*β*‐*β*	*β*‐5	
1	GVL Eucalyptus	3018	3.6	42.7	53.5	11.3	4.4	2.3	6.7
2	GVL White birch 1	3619	3.9	42.7	53.5	11.5	4.2	2.8	6.3
3	GVL White birch 2	2807^a^	2.0	36.7	61.2	13.8	4.7	3.1	6.8
4	GVL SCB 1	2956	3.6	42.1	54.3	25.3	1.5	5.0	4.9
5	GVL SCB 2	2147^a^	6.7	39.6	57.3	21.5	1.5	5.3	4.7
6	GVL Scots pine	4646	4.3	95.0	0.7	12.5	2.4	9.5	3.9
7	EtOH Eucalyptus	2466	1.4	33.2	65.3	5.6	3.2	2.4	7.0

a SEC column changed; results not directly comparable.

The thermochemolysis revealed that white birch, eucalyptus and SCB lignins had predominantly syringyl type units present at >50%. Guaiacyl type units were the next most common (33.2%–42.7%) with only minor amounts of *p*‐hydroxyphenyl units present. The GVL Scots pine presented mainly guaiacyl type units (95%) with small amounts of syringyl (0.7%) and *p*‐hydroxyphenyl (4.3%) units. The results are well in line with the literature; softwood lignins are composed almost exclusively of guaiacyl units, while hardwood lignins contain higher quantities of syringyl type units [50]. It is possible that lignin with high G content is more poorly extracted than lignin with high S content, supporting the findings during lignin extraction, where the GVL pine (high G content, softwood) had the lowest lignin yields compared to the hardwoods with high S content.

2D‐HSQC NMR was used to measure the interunit linkages in the lignin samples. The linkage amount is presented as % of aromatic units. *β*‐O‐4 linkages were the predominant linkage found in all lignin samples. The *β*‐O‐4 content can be used to calculate the maximum theoretical monomer yield; the square of the fraction of *β*‐ether bonds indicates the theoretical maximum monomer yield, assuming that only the *β*‐O‐4 linkages are cleaved during the depolymerisation [51]. According to this, the SCB with the highest *β*‐O‐4 content would produce the highest monomer yield of these lignin samples. However, the theoretical maximum monomer yield for all lignin samples remains low (0.3%–6%).

The methoxy content is expressed in mmol/g of lignin and ranged between 4–7 mmol/g, with the GVL Scots pine having the least and the EtOH eucalyptus the most. Coumaric alcohol (H unit) has no methoxy groups, coniferyl alcohol (G unit) has one methoxy group, whereas the sinapyl alcohol (S unit) has two. Softwoods such as pine generally only present 5%–10% S units, whereas hardwoods have around 50% S‐units [52]. As the S‐units have more methoxy groups, the lignins with more S‐units will also have higher methoxy content. The GVL Scots pine has only 0.7% S units and 95% G‐units, explaining the lower methoxy content.

The lignin samples were mixed with ethanol to investigate their solubility in ethanol at room temperature and to analyse how many monomers were released. After mixing overnight in ethanol, about 40 wt% of the GVL lignins and 79 wt% of the EtOH organosolv lignin were solubilised in ethanol, and less than 1 wt% of monomers were found in these dissolution experiments in all lignins (Table S4).

### 3.3 Catalyst Synthesis and Characterisation

Molybdenum‐based catalysts have gained attention in biomass valorisation as alternatives to noble‐metal catalysts. In most cases, supported molybdenum‐catalysts have been used [28, 29, 30, 31]. In this work, we chose to study unsupported molybdenum catalysts, as they are an attractive alternative to supported catalysts for processing challenging feedstocks in a slurry reactor [53]. Molybdenum carbides were chosen due to their good performance in several depolymerisation studies. As an alternative depolymerisation catalyst, we selected molybdenum phosphide, which has been studied only in a handful of reports. Three unsupported molybdenum carbides and two phosphides were prepared according to methods reported by Tang et al. [42] (MoC‐1, MoC‐2), Ma et al. [43] (MoC‐3), Stinner and Prins [44] (MoP‐1) and Cheng et al. [45] (MoP‐2) (see Supporting Information for detailed information).

Physisorption analysis revealed that all catalysts exhibited rather low surface areas (See Table S1). The carbides had a BET between 13–20 m^2^/g, and the phosphides even lower at 2–4 m^2^/g. XRD was used to identify the different crystalline phases in the catalysts. The MoC‐1 displayed mainly peaks corresponding to the cubic *α*‐MoC_1−*x*_ phase (JCPDS no. 89–2868, Figure S1a) [42]. The MoC‐2 catalyst showed a more crystalline structure with peaks corresponding to both *α*‐MoC_1−*x*_ and hexagonal *β*‐Mo_2_C (JCPDS no. 35–0787, Figure S1b). The wide peaks of the MoC‐3 indicated the presence of small MoC_*x*_, crystallites which is consistent with the results reported by Ma et al. (Figure S1c) [43]. The MoP‐2 and MoP‐1 catalysts displayed mainly hexagonal MoP phase (JCPDS 24–0771, Figure S1d,e) [44]. In addition, MoP‐2 contained small amounts of MoOPO_4_ (JCPDS 34–1276), whereas the MoP‐1 catalyst showed the presence of MoO_2_ (JCPDS 32–0671) [54].

SEM images of the MoP‐1 and MoP‐2 showed agglomerated structures, which correspond with the bigger crystallite size observed in XRD, as well as the low surface area of the catalysts (Figure S2). The MoC‐1 catalyst showed a nanosheet morphology consistent with the report by Tang et al. (Figure S3) [42]. The morphology of MoC‐3 showed clustering of the nanosized particles into bigger agglomerates. Further analysis of MoC‐1 by transmission electron microscope (TEM) confirmed the nanosheet‐like structure (Figure S4). Moreover, the images showed the existence of partly amorphous regions with bent layers among the crystalline phase.

### 3.4 Catalyst Screening

To test the performance of the prepared catalysts, a series of depolymerisation experiments was conducted using the EtOH eucalyptus lignin at 280°C. For each catalyst, experiments with and without the addition of hydrogen gas (20 bar at RT) were carried out. Low catalyst loading of 5 wt% was chosen to highlight the differences and because many examples in the literature used high loading of catalyst (typically 50 wt% of lignin) [32, 55, 56, 57]. Low catalyst loading is a more viable option for potential industrial purposes. In addition, two experiments without a catalyst were done as a reference. The length of the experiments remained constant at 4 h. After the experiments, the cooled reaction mixture was collected and filtered. The filtrate was sampled for GC–MS analysis and evaporated using a rotary evaporator, forming the low‐molecular‐weight lignin oil, which contained monomeric and oligomeric products. The solids from filtration were dissolved in THF to separate high molecular weight residual lignin from the solids. Molecular weights of the lignin oil and residual lignin were determined by SEC. However, some of the residual lignin samples were insoluble in NaOH for the SEC analysis. This was attributed to high Mw and heavily condensed structures. The remaining solids, consisting of the catalyst and char, were weighed to determine the extent of char formation. Mass balances for all experiments were mostly above 80%, which is a good level for small‐scale experimental work. Loss of different fractions could have occurred at the many stages of the sample workup when transferring between vessels. One main cause for loss could be the sample caught on the reactor vessels walls and the crevices on the reactor lid. Detailed numerical results from all the experiments are presented in Supporting Information (Table S5).

**FIGURE 4:**
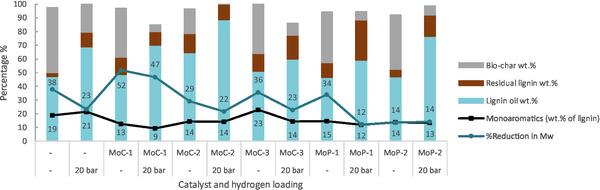
Comparison of catalysts at 5 wt% on depolymerisation of EtOH eucalyptus lignin (Conditions: 2 g lignin, 0.1 g catalyst, 80 ml EtOH, 280°C).

In the catalyst screening experiments with no added hydrogen, the lignin oil yields were moderate (46–64 wt%), and char was formed at rather high yields of 35–40 wt% (Figure 4). Lower char yield (18 wt%) was obtained only with the MoC‐2 catalyst. The reactions with 20 bar H_2_ loading gave considerably higher lignin oil yields of 60–88 wt% and lower char yields (9–20 wt%). The highest lignin oil yield (88.6 wt%) and no char formation was obtained with 20 bar H_2_ loading using the MoC‐2 catalyst, consisting of both *α*‐MoC_1−*x*_ and hexagonal *β*‐Mo_2_C phases. The residual lignin yields did not show clear trends with catalyst or hydrogen loading. Monomer yields were moderate with all catalysts, with the highest yields (22.8 wt%) obtained with the MoC‐3 catalyst without initial hydrogen pressure. Interestingly, around 20 wt% monoaromatics were obtained even without a catalyst, indicating that at these temperatures, thermal reactions also play a role in the depolymerisation. With all catalysts, monomer yields decreased slightly when hydrogen gas was loaded, compared to reactions with no hydrogen. Similar results were observed, i.e. additional H_2_ pressure was disadvantageous to monomer yields, by Ma et al. while studying molybdenum carbide catalysts in the depolymerisation of Kraft lignin [28]. In another study where nickel catalysts were applied, it was also noted that additional H_2_ did not affect monomer yields and that the alcohol solvent provided the necessary active hydrogen species [58]. The hydrogen gas may also suppress chemisorption of the ethanol over the surface of the catalyst. The effect of the H_2_ is contradictory; on the one hand, lignin oil yield is greatly improved, and char formation is minimised, while slightly smaller monomer yields are observed.

To investigate the extent of the depolymerisation, the reduction of the lignin oil Mw was calculated by comparing to the Mw of the substrate lignin (2466 Da) as described in Equation (2), which follows the approach of De Saegher et al. [59]. All lignin oils showed a reduction of Mw between 12 and 52% compared to the substrate. The largest reduction was obtained with the MoC‐1 catalyst; 47% and 52% with and without hydrogen gas, respectively. HSQC NMR analysis of the lignin oil produced with MoC‐1 in the presence of hydrogen showed the absence of *β*‐O‐4, *β*‐*β*, and *β*‐5 linkages, which were clearly visible in the EtOH eucalyptus lignin (Figure S8).

The monoaromatic products were quantified and identified by GC–MS analysis (Table S6). Due to the large number of products, the compounds were grouped in two ways: (i) based on the H/G/S‐structure and (ii) based on the side chain structure (saturated, unsaturated and oxygenated). Compared to the substrate lignin, the monoaromatics contained more syringyl‐units (up to 88% vs. 65% of the EtOH eucalyptus lignin) (Figure S9a). Furthermore, the selectivity to S‐type products was increased by the use of a catalyst and the addition of H_2_. The enrichment of S‐type monoaromatics is expected because easily cleavable *β*‐ether linkages are more prevalent among syringyl than guaiacyl type substructures [60].

For elucidation of the side chains, the structures present in H, G, and S lignin monomers were grouped into saturated, unsaturated and oxygenated side chains. Similar side chains were detected after all thermal and catalytic treatments with and without H_2_ loading; most of the side chains were saturated alkyls or ketones (Figure S9b,c). Minor amounts of unsaturated hydrocarbons were also detected in all experiments. Under these conditions, the catalyst and hydrogen addition had relatively little effect on the product distribution; selectivity to deoxygenated side chains was mostly between 55 and 62 wt%. The highest selectivity to products with deoxygenated side chains was observed with MoC‐1, up to 68 wt%.

Based on the catalyst screening experiments, high lignin oil yields were obtained with both MoC and MoP catalysts, especially in the presence of hydrogen. Of the studied carbides, MoC‐1 showed the best performance in terms of low char yield, high degree of depolymerisation and high deoxygenation activity of the side chains. Moreover, we were interested in further investigating the catalyst consisting of purely *α*‐MoC_1−*x*_ phase in the depolymerisation. Although the phosphide catalysts gave slightly lower lignin oil yields and lower reduction of Mw compared to carbides, we were intrigued to study the best performing phosphide, MoP‐2, further, due to the low number of studies on phosphides in lignin depolymerisation.

A further depolymerisation experiment was carried out using MoP‐2 at 315°C with 20 bar H_2_ loading, which resulted in 44 wt% lignin oil, 10.5 wt% monomers and a high char yield of 35 wt% (Table S5, entry 12). Interestingly, the reduction of molecular weight was 67%, and furthermore, the selectivity to products with deoxygenated side chains increased to 75 wt% (Figure S9b–c), indicating that the reaction temperature plays a significant role in the lignin depolymerisation. To prepare for scaling up the depolymerisations from the 200 ml reactor to a 1 L reactor, we performed an experiment in the same reaction conditions (315°C, 20 bar H_2_ loading and 5 wt% MoP‐2) with double the amount of lignin and ethanol (4 g and 160 ml, respectively). Notably, the lignin oil yield nearly doubled from 44 wt% to 79 wt%, and the char formation reduced from 35 wt% to only 3 wt%, when moving from the 200 mL reactor to the 1 L reactor (Table S5, entry 13). Molecular weight of the lignin oil reduced 48% in comparison to the substrate, which is less than in the smaller reactor, but much higher than observed at 280°C. Additionally, higher monomer yields (14 wt%) were observed in the larger reactor, and over 80 wt% of the monoaromatics contained deoxygenated side chains. The main difference between the two setups is the relative volume of the headspace, which results in a higher amount of H_2_ in the bigger reactor; using the ideal gas law, it was determined that the H_2_ amount was over 3 times more per gram of lignin in the 1 L reactor. The results are well in line with our previous observation on the effect of hydrogen on increasing the lignin oil and reducing char yields, and furthermore, they underline the importance of reaction temperature on the depolymerisation.

### 3.5 Depolymerisation of GVL Lignins

**FIGURE 5:**
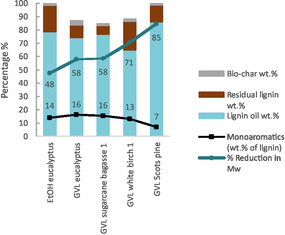
Comparison of different lignin feeds using MoP‐2 (1 L reactor: 4 g lignin, 160 ml EtOH, 5 wt% catalyst, 315°C, 20 bar H_2_).

Encouraged by the high lignin oil yield and high extent of depolymerisation in the 1 L reactor, we used the same conditions (5 wt% MoP‐2 catalyst, 315°C and 20 bar H_2_) to compare the lignins obtained by GVL fractionation from different biomasses: eucalyptus, SCB, white birch and Scots pine. Depolymerisation of the GVL eucalyptus and GVL SCB lignins gave very similar results compared to the EtOH eucalyptus; about 75 wt% yield of lignin oil with minimal char formation (Figure 5). The reduction of Mw was at 58% in both experiments, which is slightly higher than the 48% obtained with the EtOH eucalyptus. The yield of monoaromatics was 16 wt%, which is similar to the previous experiments. Interestingly, the monomer yield is unaffected by the *β*‐O‐4 content of the lignins, which ranged from 6% in the EtOH eucalyptus to 25% in the SCB lignin. The observed monomer yields are significantly greater than the theoretical monomer yields calculated based on the *β*‐O‐4 content, which suggests that linkages other than the *β*‐O‐4 have also been cleaved in the depolymerisation. Depolymerisation of the GVL white birch produced 65 wt% lignin oil with 71% reduction of the Mw and 13 wt% of monoaromatics. The highest lignin oil yield was achieved with GVL Scots pine lignin, 86 wt%. Despite the relatively low monomer yield (7 wt%), Scots pine exhibited the greatest reduction in Mw (85%) compared to the other feedstocks. Overall, in comparison with the EtOH eucalyptus lignin, the GVL lignins yielded higher amounts of lignin oil and exhibited greater reduction in Mw under the same reaction conditions. This could be explained by the higher *β*‐O‐4 content of the GVL lignins (11%–25%) compared to the EtOH eucalyptus (6%), showing that the GVL fractionation produces lignins which are well‐suited for depolymerisation.

**FIGURE 6:**
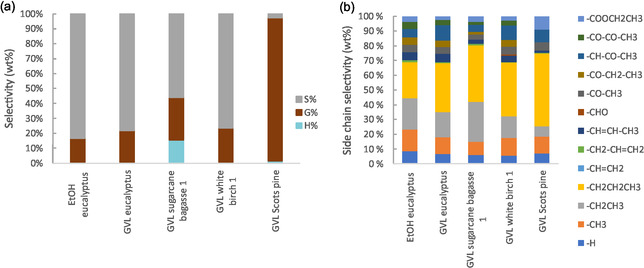
Monoaromatics selectivity to (a) H/G/S‐units and (b) different side chains in the depolymerisation of lignins in ethanol with 5 wt% MoP‐2, 20 bar H_2_ at 315°C.

According to the GC–MS analysis, the produced monoaromatics reflected the H/G/S ratios of the original lignin (Figure 6a and Table S7). The eucalyptus, SCB and white birch GVL lignins consisted primarily of S‐type units, which is also seen in the product distribution. Interestingly, the bagasse lignin also produced H‐type monomers with 15% selectivity, despite the H‐unit content being similar to the other lignins. The GVL Scots pine lignin consisted mainly of G‐type units, which are also the main units present in the lignin oil. Similarly to the catalyst screening experiments, syringyl units were more prevalent in the lignin oils compared to their content in the substrate lignins.

Analysis of the side chain structures revealed that the monoaromatics contained predominantly deoxygenated side chains with about 75–85 wt% selectivity (Figure S10, Table S7). The experiments performed in the 1 L reactor had considerably less oxygenated side chains on the monoaromatics compared to the experiments in the smaller reactor, which is mostly attributed to the higher reaction temperature. However, comparing the depolymerisation of EtOH eucalyptus lignin in the two reactors at 315°C also shows a difference; the selectivity to deoxygenated side chains increased from 75% to 81% in the 1 L reactor, which can be explained by the increased availability of H_2_ aiding hydrodeoxygenation of the oxygenated structures. When taking a closer look at the identity of the side chain structures, it is seen that the propyl side chain is more abundant in the GVL lignin oils compared to the EtOH eucalyptus (Figure 6b). The GVL eucalyptus, SCB and white birch lignins show 22%–27% selectivity to 4‐propylsyringol, and in the case of Scots pine, 4‐propylguaiacol is produced with 49% selectivity.

### 3.6 Effect of Depolymerisation Conditions

**FIGURE 7:**
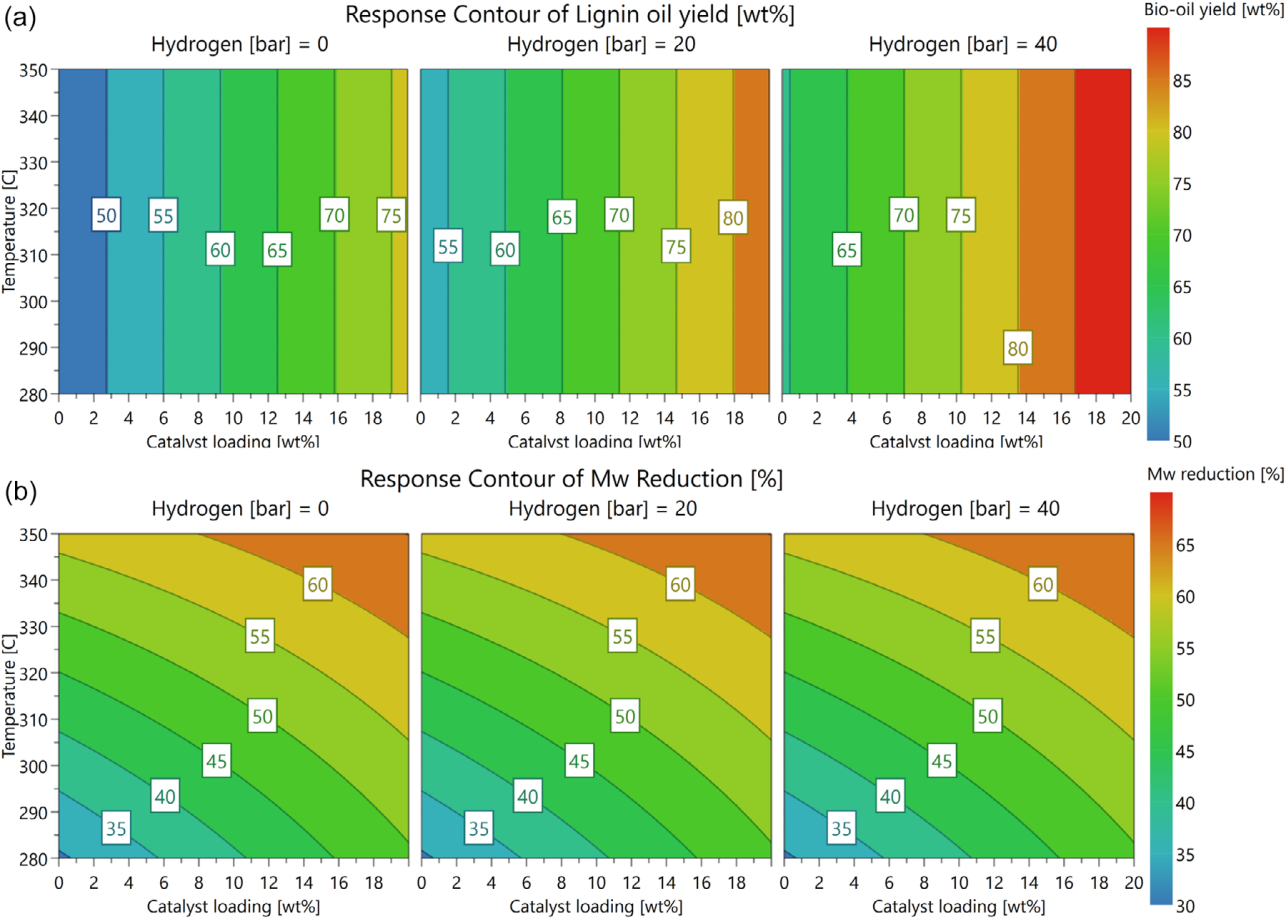
Response surfaces for (a) lignin oil yield and (b) reduction of lignin oil molecular weight based on the statistical analysis.

The previous experiments indicated that the catalyst, hydrogen loading and reaction temperature all have a significant effect on depolymerisation. To systematically study the effect of the depolymerisation conditions, both on the product yields and properties, a design of experiments study was conducted using a full factorial design with 11 experiments, including three repetitions. For the catalyst, we chose MoC‐1, consisting of *α*‐MoC_1−*x*_, which gave the highest molecular weight reduction along with good lignin oil yields in the catalyst screening tests. In the previous experiments with MoP‐2, increasing the depolymerisation temperature resulted in lower Mw of the lignin oil, and therefore the studied temperature range was expanded from 280°C up to 350°C. Similarly, the range of hydrogen loading (0–40 bar at RT) and catalyst amount (0–20 wt%) was increased from the previously used. The responses analysed were the yields of monomers, lignin oil and char. In addition to the yields, reduction of the lignin oil Mw and selectivity to deoxygenated side chains in the monomers were analysed. Furthermore, the amount of aliphatic hydroxyl groups, determined by ^31^P‐NMR, was selected for the analysis. Out of the different feedstocks studied in the GVL fractionation process, white birch was selected as the most promising based on the high‐quality pulp fraction. Scaling up the fractionation yielded GVL white birch lignin 2, which was used as feedstock for the DoE experiments. The experiments were performed in the 200 ml autoclave to minimise the quantities of lignin and catalyst needed. The results were fitted using MODDE software version 13.0.2 using multiple linear regression (MLR) (Figures S11 and S12). Response surfaces based on the statistical models are shown in Figures 7 and S13.

The yields of lignin oil and char varied between 35–92 wt% and 0–43 wt%, respectively, which are in a similar range as in the previous experiments (Table S8). Based on the statistical analysis, the use of a catalyst and hydrogen gas had a positive effect on the lignin oil yield and a negative effect on the bio‐char yield (Figure 7a and Figure S13). Moreover, increasing the temperature increased the char formation, especially in the absence of a catalyst and hydrogen. Monomer yields of 7–12 wt% were observed, the yields being slightly lower than with the eucalyptus lignin. Such a small variation did not enable a reliable statistical analysis of the parameters affecting the monomer yields. However, the selectivity towards deoxygenated side chains was clearly affected by the catalyst and conditions; the selectivity increased from 61 to 99.9 mol% by increasing the temperature and by using hydrogen gas and a catalyst. While temperature had the strongest positive effect, it is evident that the monomer selectivity can be tuned by the catalyst and reaction conditions. The lignin oil properties were also heavily affected by the reaction parameters. Based on SEC analysis, the reduction of the lignin oil molecular weight was in the range 24%–64% compared to the starting GVL white birch 2 at 2800 Da. It should be noted that these samples were measured after a change of the SEC column, so the Mw values are not directly comparable to the previous lignin oil samples (Table S5). Temperature had the strongest decreasing effect on the Mw (Figure 7b). Very interestingly, we also observed that the depolymerisation conditions had a clear effect on the amount of aliphatic hydroxyl groups in the produced lignin oils. At high temperature, hydrogen pressure and catalyst loading, the aliphatic hydroxyls amounted to 1 mmol/g, whereas without hydrogen, the value decreased to less than 0.3 mmol/g from the feedstock value of 2 mmol/g (Table S9). These findings indicate that hydrogen is essential to depolymerise lignin efficiently to lignin oil and monomers, supporting that H_2_ stabilises reactive intermediates, thus avoiding repolymerisation [25]. Moreover, the catalyst has a crucial role in increasing the lignin oil yield and suppressing char formation, which both underline the importance of the catalyst in stabilising the reactive fragments released during the depolymerisation (Figure S12). Activity of the catalyst is further underlined by its positive effect on the side chain deoxygenation and reduction of Mw. In summary, these results show how to optimise the depolymerisation conditions, not only to maximise the lignin oil yield but also how to tune the properties of the resulting products to meet specific application needs.

To see how the DoE results from white birch depolymerisation compare to another feedstock, two of the most promising reaction conditions were chosen to test on SCB lignin. In two experiments, GVL SCB 2 was depolymerised in the presence of 20 wt% MoC‐1 and 40 bar H_2_ at 280°C and at 350°C. At 280°C, 76 wt% of lignin oil was formed with 8.7 wt% of monomers, and the reduction of Mw was at 38% (Figure S15a). The experiment at 350°C gave a better lignin oil yield (93 wt%) and monomer yield (11.6 wt%) compared to the lower temperature. Additionally, the lignin oil Mw was reduced to 55%. Even though temperature had a more pronounced effect on the lignin oil yield in the case of SCB lignin compared to white birch, the results were well in line with the DoE study, and high yields of lignin oil with minimal char production were obtained. In a further experiment, the lignin to ethanol ratio could be doubled without negatively affecting the product yields, which is promising for future upscaling of the process (Figure S15b).

## 4 Conclusions

This work establishes *γ*‐valerolactone (GVL) organosolv fractionation as an efficient route for producing high‐quality lignins from diverse biomass sources, enabling their subsequent catalytic depolymerisation into monophenolics and low‐molecular‐weight lignin oils. The fractionation produced over 90% yields of high‐purity lignin fractions, particularly from hardwoods and herbaceous biomass, with molecular weights ranging from 2100 to 4600 Da. The GVL lignins were depolymerised using unsupported molybdenum‐based catalysts under reductive conditions in ethanol; to our knowledge, this is the first study reporting non‐noble metal catalysed depolymerisation of GVL lignins. High yields of low‐molecular‐weight lignin oil (up to 92 wt%) were obtained both with molybdenum carbide and molybdenum phosphide catalysts. The aromatic monomer yields ranged from 7 to 16 wt%. Importantly, the depolymerisation conditions, catalyst, hydrogen loading, and temperature, were shown to be critical both for maximising the yield of the lignin oil and for modifying the properties of the produced lignin oil and monoaromatics. This opens up the possibility to tune the catalytic depolymerisation process towards the targeted application. Our findings demonstrate that GVL lignins are versatile feedstocks for sustainable chemical and material production, supporting the development of integrated biorefinery concepts where all major lignocellulosic fractions are valorised.

## Supporting Information

Additional supporting information can be found online in the Supporting Information section. The authors have cited additional references within the Supporting Information [61, 62]. **Supporting** **Fig.** S**1:** XRD diffractogram a) MoC‐1, b) MoC‐2, c) MoC‐3, d) MoP‐1 and e) MoP‐2. **Supporting Fig. S2** **:** SEM image of a) MoP‐1 and b) MoP‐2 catalyst with 8 kV acceleration voltage at 1000 x magnification. **Supporting Fig. S3** **:** SEM images of MoC‐1 catalyst with 2 kV acceleration voltage at a) 1000 x and b) 10 000 x magnification and c) MoC‐3 at 1000 x magnification. **Supporting Fig. S4** **:** TEM images of MoC‐1. **Supporting Fig. S5** **:** Mass flows of the GVL fractionation of white birch. **Supporting Fig. S6** **:** Maximum furfural yield achieved. **Supporting Fig. S7** **:** Process flow of sample treatment. **Supporting Fig. S8** **:** HSQC NMR spectra of EtOH eucalyptus lignin and the depolymerised lignin oil formed under conditions of 280°C, 20 bar H_2_, 5 wt% MoC‐1 catalyst. **Supporting Fig. S9** **:** GC‐MS results of monomers in the catalyst screening experiments. Reaction conditions: 2 g EtOH eucalyptus lignin, 80 mEtOH, 280°C, 5 wt% catalyst. a) H/G/S ratio of monomers. b) Side chain groups on monomers, saturated, unsaturated and oxygen in the side chain. c) Type of side chain on monomers. **Supporting Fig. S10:** GC‐MS results of monomers in different feedstock experiments. Reaction conditions 315°C, 5 wt% MoP‐2 catalyst, 20 bar H_2_. Side chain groups on monomers, saturated, unsaturated and oxygen in the side chain. **Supporting Fig. S11:** Observed vs. predicted values for a) yield of monomers, lignin oil and char and b) aliphatic hydroxyls, selectivity to deoxygenated side chains and reduction of Mw. **Supporting Fig. S12:** Coefficients for a) yield of monomers, lignin oil and char and b) aliphatic hydroxyls, selectivity to deoxygenated side chains and reduction of Mw. **Supporting Fig. S13:** Response surfaces for the yield of a) monomers and b) char. **Supporting Fig. S14:** Response surfaces for a) selectivity to deoxygenated side chains and b) amount of aliphatic hydroxyls. **Supporting Fig. S15:** Depolymerisation of GVL lignins. a) SCB depolymerisations using 2 g lignin, 80 ml EtOH, 20 wt% MoC‐1and 40 bar H2. b) Depolymerisation of GVL white birch 2 with higher lignin concentration. Reaction conditions: 4 g lignin, 80 ml EtOH, 10 wt% MoC‐1, 40 bar H_2_. **Supporting Table S** **1:** BET surfaces areas of prepared catalysts. **Supporting** **Table** **S2:** Composition of biomass feedstocks used in GVL fractionation. **Supporting** **Table** **S3:** Composition of GVL lignins. **Supporting** **Table** **S4:** Lignin dissolution, 1 g lignin, 40 ml EtOH, room temperature, overnight. **Supporting** **Table** **S5:** Reaction conditions, yields of lignin oil, residual lignin, biochar and monoaromatics. Molecular weights of EtOH and THF soluble fractions. **Supporting** **Table** **S6:** Monoaromatics (wt%) of depolymerisation of EtOH eucalyptus lignin in ethanol (2 g lignin, 5 wt% catalyst, 80 ml EtOH). **Supporting** **Table** **S7:** Monoaromatics (wt%) of depolymerised GVL lignin. **Supporting** **Table** **S8:** Experimental data for DoE experiments. **Supporting** **Table** **S9:** ^31^P‐NMR results for DoE experiments of white birch depolymerisation. Results are given as mmol/g.

## Conflicts of Interest

The authors declare no conflicts of interest.

## Supporting information

Supplementary Material

## Data Availability

The data that support the findings of this study are available in the supplementary material of this article.
