# Supplementary material for: Depolymerisation of γ‐Valerolactone Organosolv Lignins with Unsupported Molybdenum‐Based Catalysts
Source: ChemSusChem. 2026 Feb 8;19(3):e202500643. doi: 10.1002/cssc.202500643 (PMC12883147; doi:10.1002/cssc.202500643)
Supplement: Supplementary file 1 — Supplementary Material [file CSSC-19-e202500643-s001.pdf]

Supporting Information

Depolymerisation of GVL Organosolv Lignins with Unsupported Molybdenum-based Catalysts

Silja Känsäkoski,[a] Saravanan Kasipandi,[a†] Taina Ohra-aho,[a] Tom Wirtanen,[a] Juha Lehtonen,[a] David Martin Alonso,[b] Francisco Vila,[b] Sari Rautiainen[a*]

[a] *VTT Technical Research Centre of Finland, P.O.Box 1000, FI-02044, VTT, Finland*

[b] *EQS Group (Sustainable Energy and Chemistry Group), Institute of Catalysis and Petrochemistry (CSIC), C/Marie Curie 2, 28049 Madrid, Spain*

† Deceased April 2024.

**E-mail: sari.rautiainen@vtt.fi*

1. Detailed Experimental Section

1.1. Materials

Reagents were commercially purchased and used as such without further purification. Ethanol (Altia, ≥99.5%), tetrahydrofuran (VWR chemicals, ≥99% stabilized ACS), ammonium heptamolybdate tetrahydrate $(NH_4)_6Mo_7O_{24}·4\ H_2O$ (VWR chemicals, 81.0-83.0%), diammonium hydrogen phosphate $(NH_4)_2HPO_4$ (Merck, ≥99% ACS reagent grade), citric acid monohydrate (VWR chemicals, ≥99% ACS), 1-cyanoguanidine (Sigma-Aldrich, ≥98.0%), 2,4,6-triamino-1,3,5-triazine (Sigma-Aldrich, 98.5 - 101.5 %), cyanuric acid (Sigma-Aldrich, ≥98.0%), phenol (Sigma-Aldrich, 99%), o-cresol (Sigma-Aldrich, 99.5%), m-cresol (VWR chemicals, 99%), 4-ethylphenol (Sigma-Aldrich, 99%), 4-propylphenol (Aldrich Chemistry, 99%), 2-propylphenol (Aldrich, 98%), guaiacol (Sigma-Aldrich, 99%), 4-methylguaiacol (Sigma-Aldrich, 99%),4-ethylguaiacol (SAFC, 98%), 4-propylguaiacol (Sigma-Aldrich, 99%), eugenol, (Sigma-Aldrich, 99%), isoeugenol (Sigma-Aldrich, 98%), vanillin (Fluka AG, 99%), syringol (Thermo Fisher Scientific, 99%), 4-methylsyringol (Sigma-Aldrich, 97%), N_2 (Woikoski, 99.999%), H_2 (Woikoski, 99.999%), 1-butanol (Sigma-Aldrich, 99.8%). EtOH organosolv lignin was obtained from Fraunhofer prepared by an ethanol-water organosolv process.

1.2. Lignin separation

GVL lignin samples were produced from 4 different feedstocks, two hardwoods (white birch and eucalyptus globulus), one softwood (pinus sylvestris) and one agricultural residue (sugarcane bagasse). Woody feedstocks were used in the form of chips (approximately 2 × 2 × 0.5 cm). The sugarcane bagasse sample was crushed (without grinding to powder). All feedstocks were air dried to a 5-10 wt% moisture content. In a typical fractionation experiment, 150-200 g of biomass and 600-800 g of a liquid solution of GVL/water 70/30 by weight and 0.1 M sulfuric acid were added to a 1 L reactor and heated to the reaction temperature (125 or 130 °C) for 60-90 minutes. At the end of the reaction, the liquid was separated from the solid using vacuum filtration. The remaining cellulose was washed with hot fresh GVL/water solution. The cellulose was then washed with hot water and analysed following the NREL/TP-510-42618 protocol to determine the amount of hemicellulose and lignin extracted into the liquid phase. The liquid fraction was analysed by High Performance Liquid Chromatography (Waters 2695 system with a Bio-Rad Aminex HPX-87H column) equipped with an RI detector. Cellulose yield is calculated based on the original weight of dry wood, while hemicellulose and lignin extraction yields are calculated based on their content in the feedstock.

For the scale-up reactions we used a 20 L recirculation reactor built by EDIBON. The biomass (1-2 kg dry) was placed in an acid-resistant perforated basket and introduced inside the reactor. The liquid (GVL/water 70/30 0.1 M sulfuric acid) was heated to the reaction temperature by an electrical heater situated in the recirculation loop. At the end of the reaction, the liquid was drained from the bottom of the reactor by applying some pressure. After that the cellulose was washed inside the reactor with hot GVL/water and finally with hot water. The analytical procedure was similar to the small-scale samples.

To precipitate the lignin from the liquid fraction, water was added to a ratio water/GVL = 8 and the solution was centrifuged at 4000 rpm. The lignin was washed with hot water until the GVL and carbohydrates content was <0.5 wt% and air dried. The chemical composition of the lignin was analysed following the NREL/TP-510-42618 protocol. After precipitating the lignin, the aqueous solution was concentrated and the C5 sugars were converted to furfural using a continuous flow reactor at 225°C.[1]

1.3. Lignin analysis

See section 1.7 for details on SEC.

For the analysis of lignin H/G/S –ratio, thermochemolysis with tetramethyl ammonium hydroxide (TMAH) was performed using a filament pulse pyrolyser (Pyrola 2000 from Pyrol AB, Sweden) connected to gas chromatography with a flame ionisation detector (Agilent 7890B) and mass selective detector (Agilent 5977A). Thermochemolysis was performed at 600 °C for 2 seconds after mixing dried solid sample with TMAH reagent. Formed methyl derivatives were separated by GC having a mid-polar capillary column coated with a stationary phase of 14% cyanopropyl-phenyl 86% dimethyl polysiloxane (DB-1701, 30 m × 0.25 mm, film 1 µm) and detected by FID and MSD with mass scan range of m/z between 30 and 600 (EI 70 eV). Identification of methylated S, G, and H type lignin derivatives was performed according to the previous studies.[2] For the determination of S/G/H ratios peak areas of methylated lignin derivatives were integrated and relative molar abundances were calculated.

Amounts of aryl ether (β-O-4), phenyl coumaran (β-5), and resinol (β-β) type interunit linkages were quantified from the lignin samples using ^{1}H-^{13}C HSQC (hsqcetgpsisp2.2) NMR spectroscopy based on the literature assignments.[3] The NMR analyses were performed using a Bruker AVANCE III 500 NMR (11.7 T) spectrometer with a broadband optimised 5 mm probe head at 298 K. The spectra were recorded from different samples at concentration of 130 mg/ml and referenced to residual (^{1}H, 2.50 ppm) and deuterated signal (^{13}C, 39.52 ppm) of d_6-DMSO that was used as a solvent. The acquisition time was 0.2 s and relaxation delay between the successive scans was set to 1 s. The number of scans was 192 for each 256 increments in the indirectly detected dimension. The indirectly detected dimension was zero-filled to 1024 points and Gaussian apodization was applied to both dimensions. The spectra were normalised to the externally quantified methoxy-peak, which was used together with G/S ratio for calculating the total amount of aromatic units. This method was chosen for obtaining better accuracy compared to the use of volume integral of guaiacyl C2-H2 correlation peak, when calculating the ratio between linkages and aromatic units.

The methoxy group content was determined by a headspace GC (Agilent7697A/Agilent7690B) equipped with electron capture detector (HS-GC/ECD) following the method by Baker with some modifications.[4]

1.4. Catalyst synthesis

Catalysts MoC-1 and MoC-2 were prepared by dissolving ammonium heptamolybdate (0.5 mmol), cyanuric acid (10 mmol) and melamine (10 mmol) in 50 mL deionised water and stirred vigorously for 15 hours. The milky solution was then dried at 80 °C resulting in a white powder. The powder was heated in a half-covered alumina boat in N2 atmosphere from room temperature to 450 °C at a rate of 2 °C/min and kept at that temperature for 3 hours. This was followed by pyrolyzing the material at 650 °C (MoC-2) or 750 °C (MoC-1) from room temperature (under 3 °C/min) for 3 hours.[5] The third molybdenum carbide (MoC-3)[6] was prepared by vigorously stirring dicyanamide (30 g) and 225 mL deionized water at 90 °C before adding ammonium heptamolybdate (15 g). The water was evaporated at 80 °C for 48 hours. The remaining solid was further dried at 100 °C. The solid was then pyrolyzed by heating to 450 °C (2 °C/min) and held for 1 hour then heated to 750 °C (2 °C/min) and held for 3 hours.

For molybdenum phosphide, MoP-1, 12 grams of ammonium heptamolybdate and 9 grams of diammonium hydrogen phosphate were dissolved in 90 ml deionised water.[7] The solution was aged in a round bottom flask on a heat-on block at 90 °C while vigorously stirring until the water had evaporated. The remaining solid was further dried in an oven at 120 °C for 24 hours. The solid was ground slightly prior to being calcined at 500 °C for 5 h (600 °C/h heating rate). The solid turned dark blue in color. To convert the blue powder to active metal phosphide, the catalyst was reduced in a fixed bed reactor with H_2 flow rate 160 ml/min and the powder was heated first to 300 °C (5 °C/min), then heated to 650 °C (1 °C/min) which was then held for 2.5 hours. This was followed by cooling to room temperature and to prevent bulk oxidation, the surface was passivated under

a flow of 1% O_2/N_2 for 2 hours. The second molybdenum phosphide, MoP-2, followed the same method as the MoP-1, with the addition of 13.1 grams of citric acid to the deionised water along with the ammonium heptamolybdate and diammonium hydrogen phosphate.[8]

1.5. Catalyst characterisation

Nitrogen physisorption using a Micromeritics 3Flex system was used to determine the BET surface area of the catalysts. The catalysts were first dried at reduced pressure at 200 °C for 58 h. Then the adsorption/desorption measurement was completed in liquid nitrogen, temperature of -196 °C. The BET surface area was then calculated from the adsorption isotherm using the relative partial pressure P/Po= 0.001-0.3.

X-ray diffraction (XRD) analysis was performed to determine the molecular structure and composition using PANalytical X'Pert Pro MPD with CuKα (1.5419 Å) radiation and focusing optics with programmable divergence and antiscatter slits set to 10 mm irradiated length.

Scanning electron microscopy (SEM) was performed using a Carl Zeiss Merlin electron microscope to analyse the microscopic structure and morphology. The acceleration voltage was set between 2-8 kV for the measurements. Transmission electron microscopy (TEM) was done using ThermoFisher Scientific Talos F200X G2 Scanning Transmission Electron Microscope operated at 200 kV and equipped with the four SDD Super-X energy dispersive spectrometer (EDS) detectors. The morphology and crystallinity of catalyst were investigated with S/TEM images and selected area electron diffraction (SAED).

1.6. Depolymerisation experiments

The batch experiments were performed in a 200 mL Büchi high pressure autoclave reactor (Hastelloy) and in a 1 L Autoclave engineers autoclave equipped with an inner tube (stainless steel 316). In a typical run, the reactor was charged with lignin (2-4 grams), catalyst (0-20 wt%), and ethanol (80-160 mL). The air in the reactor was purged three times with nitrogen then pressure tested. The reactor was purged another three times with hydrogen and pressurised to desired pressure with hydrogen (0-40 bar H_2) at ambient temperature. The mixture was heated to required temperature (280 °C, 315 °C, 350 °C). After reacting for 4 hours with 600 RPM stirring, the reactor was cooled to room temperature. The reaction mixture was collected and filtered, and ethanol was evaporated from the filtrate producing the ethanol soluble lignin oil. The remaining solid after filtration was dissolved in THF, then filtered and THF was evaporated using rotary evaporator to isolate the residual lignin. The remaining solid consisted of catalyst and char, and the char yield was calculated by subtracting catalyst mass from the solid residue. The mass of each fraction was recorded and compared to initial lignin mass loaded to the reactor to determine the weight percentage of the fractions. Figure S7 displays the process flow for the sample treatment.

1.7. Product analysis

Monomer yield was determined from the ethanol filtrate by using Agilent 7890A gas chromatograph combined with an Agilent 5977B mass selective detector (GC/MSD). Separation of compounds was achieved using a J&W HP-INNOWax high polarity fused silica capillary column (length: 60 m, inner diameter: 0.25 mm and film thickness: 0.25 µm) with a carrier gas (Helium) flow of 1.2 ml/min. The oven temperature program was as follows: 60 °C initial temperature was held for 1 min, then the oven was heated to 230 °C at a rate of 3 °C/min and held at this temperature for 30 min. Detection of compounds was done with mass scan range of m/z between 27 and 300 (EI 70 eV). For quantification, calibration solutions of phenolic compounds including phenol, o-cresol, m-cresol, 2-ethylphenol, 2-propylphenol, 4-propylphenol, guaiacol, 4-methylguaiacol, 4-ethylguaiacol, 4-propylguaiacol, eugenol, isoeugenol, vanillin, syringol and 4-methylsyringol were prepared, respectively. Other phenolic compounds present in the sample were calculated using 4-propylguaiacol calibration.

Size exclusion chromatography (SEC) was performed on both the lignin oil and residual lignin fractions to determine the molar masses, following the method reported by Jääskeläinen et al.[9] The samples were dissolved in 0.1 M NaOH and filtered (0.45 µm). The molar mass measurements were executed with SEC using 0.1 M NaOH eluent (pH 13, 0.5 ml/min, T = 25 °C) and PSS MCX 1000 & 100000 Å columns. Detection of elution curves were done using a Waters 2998 Photodiode Array detector at 280 nm. The molar mass distributions (MMD), weight average molar mass (Mw), number average molar mass (Mn), and polydispersity (PD) were calculated against polystyrene sulphonate standards (eight standards with a range of 3420–148500 g/mol) and using Waters Empower 3 (Milford, MA, USA) software. The lignin oil samples dissolved well in the NaOH, but some of the residual lignin samples (THF soluble), would not fully dissolve in the NaOH, hence the results are not shown for them. This could be due to highly condensed and high Mw structures. To determine the reduction in Mw during depolymerisation the Mw of lignin feedstock before ($Mw_{feedstock}$) and Mw of the product lignin oil ($Mw_{lignin\ oil}$) were used to calculate the change:

$$\%reduction\ in\ Mw = \frac{Mw_{feedstock} - Mw_{lignin\ oil}}{Mw_{feedstock}} \times 100\%$$

^{31}P NMR measurements are based on the method developed by Granata and Argyropoulos.[10] For the analyses, each sample was weighed and dissolved in N,N-dimethylformamide. After complete dissolution of lignin was achieved, $Cr(acac)_3$, pyridine, and internal standard solution (ISTD, endo-N-hydroxy-5-norbornene-2,3-dicarboximide) were added. Phosphitylation reagent (2-chloro-4,4,5,5-tetramethyl-1,3,2-dioxaphopholane) was added dropwise. Lastly, $CDCl_3$ was added to the solution resulting in clear brown to black solution. Samples were measured at room temperature directly after preparation with Bruker 500 MHz NMR spectrometer. The chemical shift (ppm) ranges for the hydroxyl groups were as follows: aliphatic OH (150-145 ppm), condensed PhOH (145-140.5 ppm), guaiacyl (140.5-139.5 ppm), catechols (139.5-138.5 ppm), *p*-OH-phenyl (138.5-137 ppm) and carboxylic acid (136-134 ppm).

2. Catalyst characterisation

2.1. BET

Table S1. BET surfaces areas of prepared catalysts

Sample	BET (m^2/g)
MoC-1	15.0
MoC-2	18.2
MoC-3	13.5
MoP-1	3.6
MoP-2	2.8

2.2. XRD

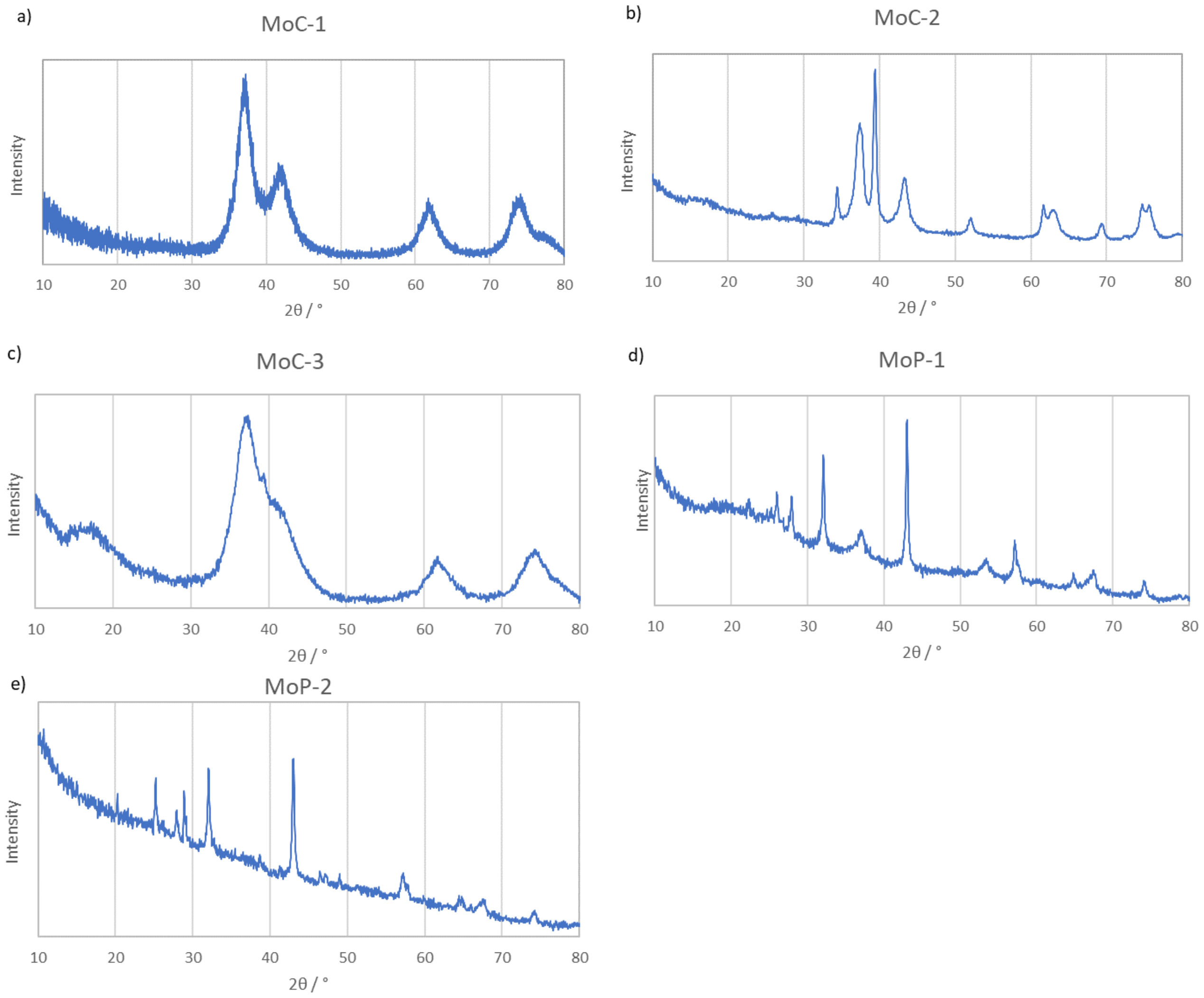

Figure S1. XRD diffractogram a) MoC-1, b) MoC-2, c) MoC-3, d) MoP-1 and e) MoP-2.

2.3. SEM

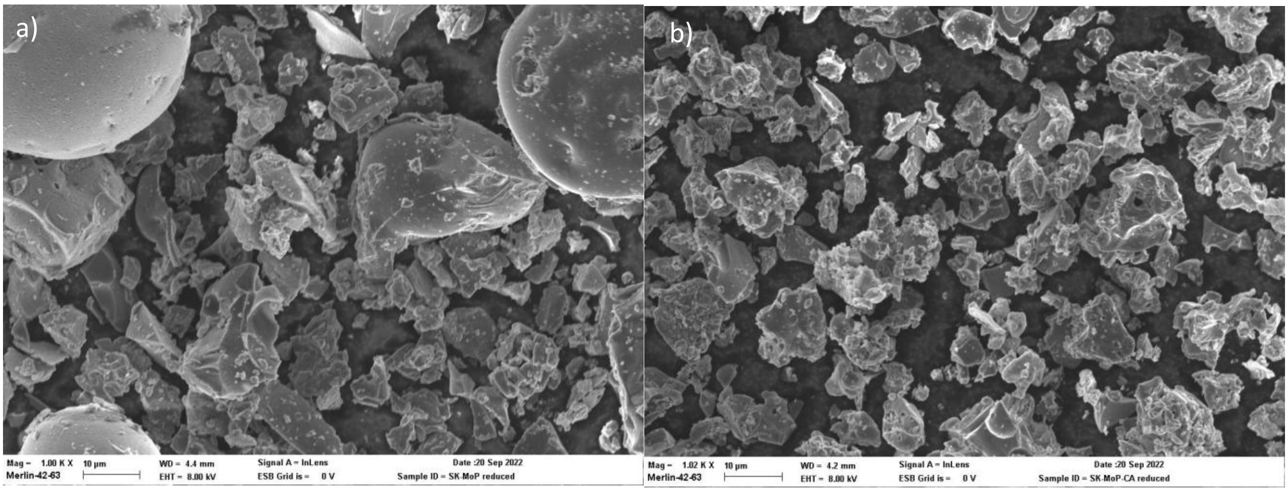

Figure S2. SEM image of a) MoP-1 and b) MoP-2 catalyst with 8 kV acceleration voltage at 1000 x magnification.

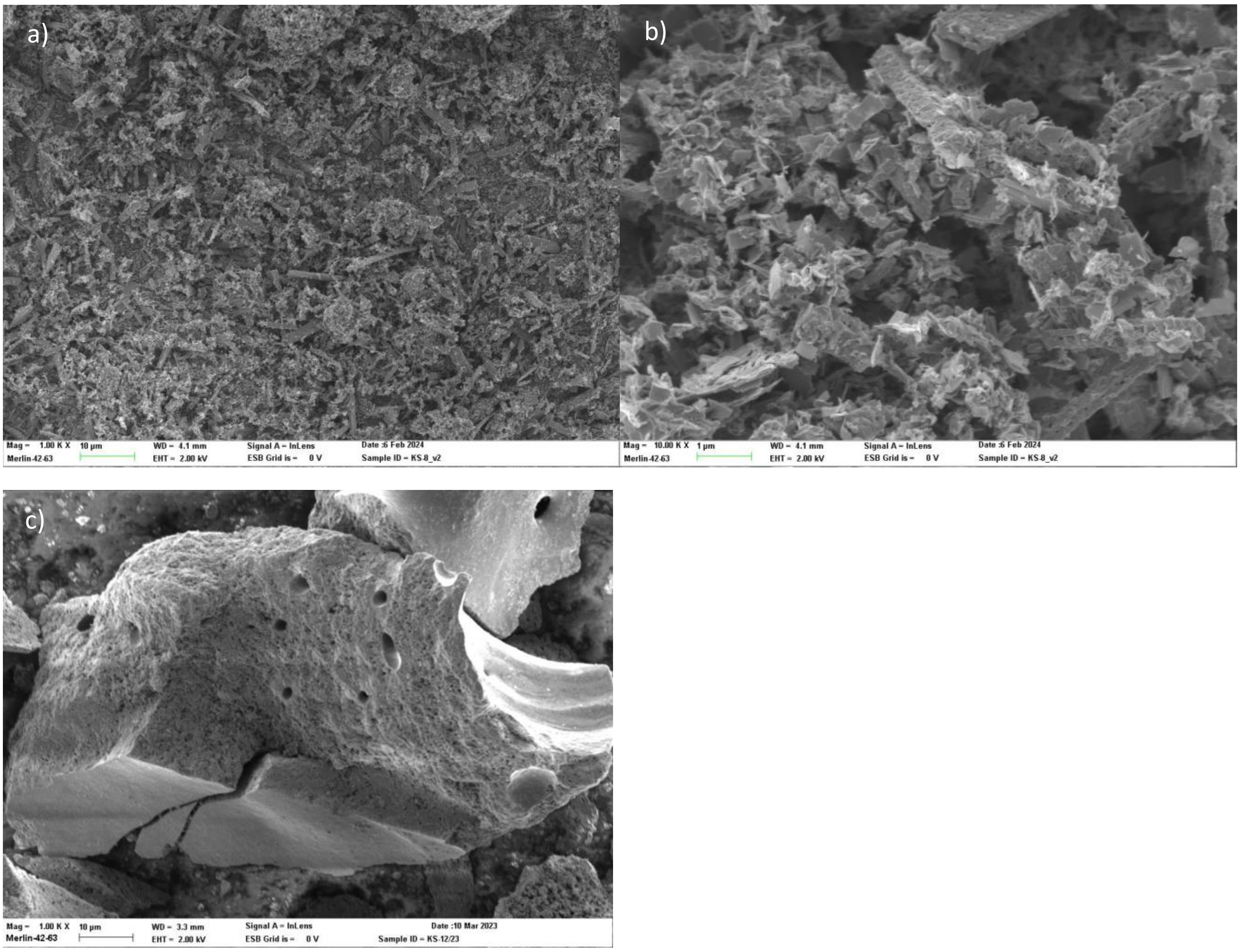

Figure S3. SEM images of MoC-1 catalyst with 2 kV acceleration voltage at a) 1000 x and b) 10 000 x magnification and c) MoC-3 at 1000 x magnification.

2.4. TEM

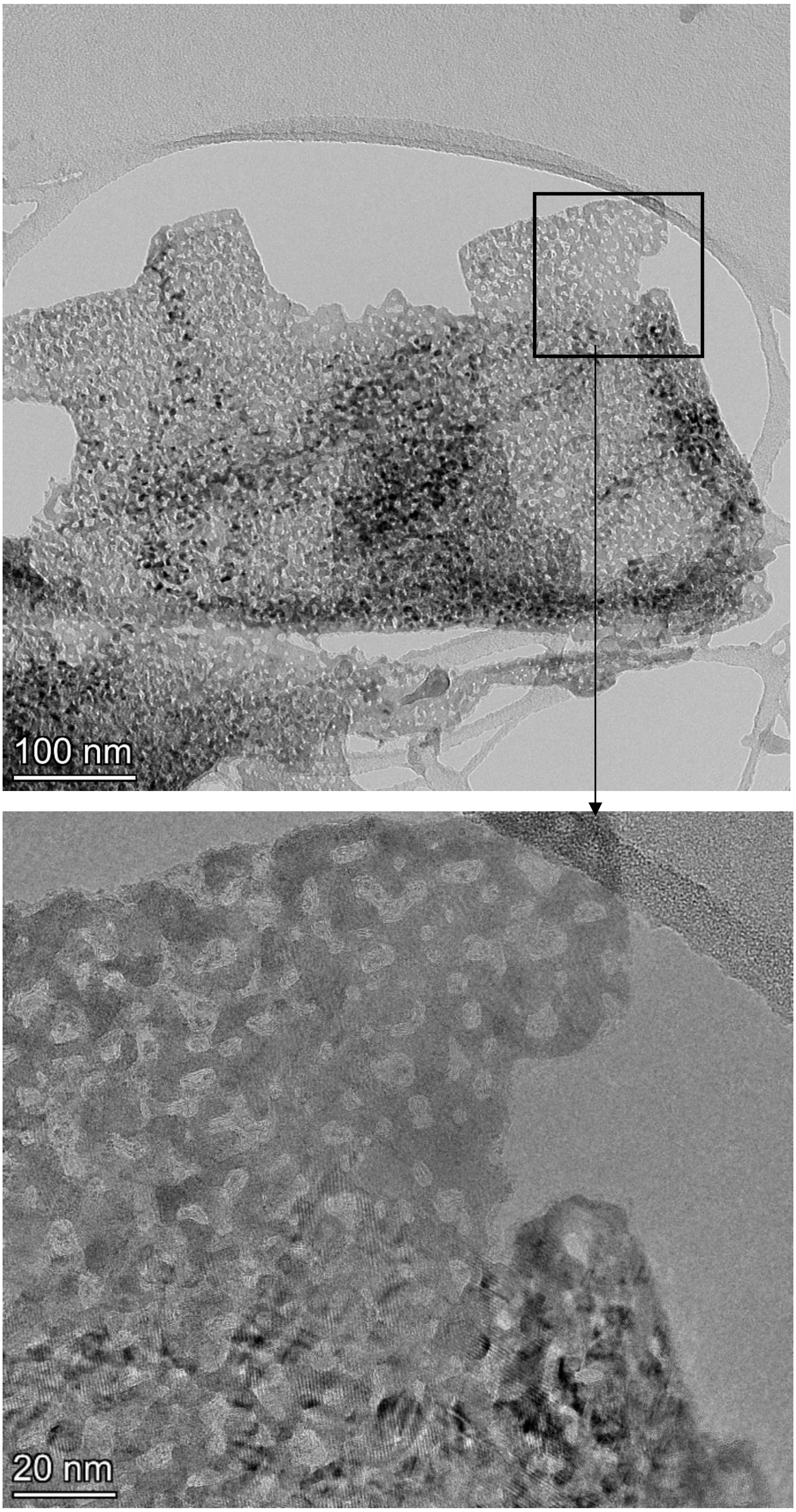

Figure S4. TEM images of MoC-1.

3. Biomass composition

Table S2. Composition of biomass feedstocks used in GVL fractionation.

	Eucalyptus	White birch	Scots pine	Sugarcane bagasse
Moisture (wt%)	6.33%	5.77%	7.49%	6.84%
Dry basis composition (wt% of biomass)				
Mass balance	97.71%	94.96%	92.79%	100.38%
Glucan (C6, cellulose)	44.53%	37.44%	42.77%	43.39%
Galactan (C6, hemicellulose)	2.00%	1.83%	2.34%	0.80%
Mannan (C6, hemicellulose)	0.91%	1.40%	12.70%	0.44%
Xylan (C5, hemicellulose)	16.49%	23.38%	6.64%	23.96%
Arabinan (C5, hemicellulose)	0.68%	0.39%	1.84%	1.61%
Acetic Acid	5.43%	5.12%	0.73%	4.47%
Extractives	0.43%	1.80%	2.20%	0.43%
Lignin (Klason + ASL)	26.80%	23.30%	28.10%	22.80%
Klason	22.70%	19.60%	27.70%	20.90%
ASL	4.10%	3.70%	0.40%	1.90%
Ash	0.44%	0.30%	0.29%	2.50%
Total C6 sugars	47.44%	40.67%	57.81%	44.64%
Total C5 sugars	17.17%	23.77%	8.48%	25.57%
Lignin + Ash + Insolubles	27.24%	23.60%	28.39%	25.30%

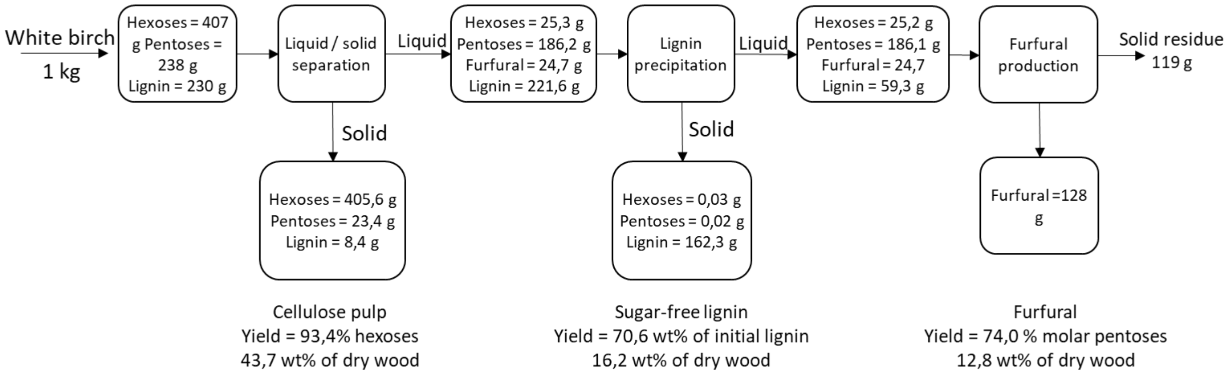

Figure S5. Mass flows of the GVL fractionation of white birch.

4. Characterisation of lignins

Table S3. Composition of GVL lignins.

Lignin	Overall yield klason lignin (wt%)	C6 sugars	C5 sugars	GVL	Ash
GVL White birch 1	82.8	0.02	0.02	0.23	0.12
GVL White birch 2	80.5	<0.5	<0.5	<0.5	0.09
GVL Eucalyptus	84.8	0.02	0.01	0.34	0.17
GVL Sugarcane bagasse 1	81.5	0.02	0.01	0.13	0.13
GVL Sugarcane bagasse 2	86.6	<0.5	<0.5	<0.5	0.13
GVL Scots pine	48.5	0.02	0.01	0.03	n.d

To analyse the solubility and monomer content of the obtained lignins, samples were dissolved in ethanol at room temperature and stirred overnight. This was executed by mixing 1 g of the lignin in 40 ml of EtOH overnight. The EtOH organosolv lignin dissolves well at room temperature, resulting in nearly 80 wt% of the lignin to be dissolved as lignin oil (Table S4). However, very few monomers are released, 0.2 wt% of the lignin. The GVL lignins did not dissolve as well, resulting in around 40 wt% of ethanol soluble fraction. Less than 1 wt% of monomers were released from the lignin samples at room temperature.

Table S4. Lignin dissolution, 1 g lignin, 40 ml EtOH, room temperature, overnight

Lignin	EtOH soluble wt%	THF soluble wt%	EtOH & THF insoluble wt%	Monomer yield (wt% of lignin)	EtOH soluble Mw (Daltons)
EtOH organosolv	79.1	17.1	1.9	0.2	1734
GVL eucalyptus	41.0	15.0	21.7	-	1694
GVL sugarcane bagasse 1	39.6	12.9	39.6	0.9	1402
GVL white birch 1	40.7	24.5	23.8	0.5	1607
GVL Scots pine	44.7	11.4	41.8	0.1	1658

5. Valorisation of carbohydrate fractions

The cellulose fraction of sugarcane bagasse and pine suffered degradation during the GVL fractionation, and consequently the synthesis of levulinic acid from the cellulose was studied. Using 50/50 GVL/water as solvent, which was the solvent used to wash the cellulose, and 0.15 M sulfuric acid as catalyst, at 165°C a 50% yield to levulinic acid could be achieved for pure cellulose, SCB-derived cellulose and pine-derived cellulose at 15wt% solids loading after 6 h of reaction. By optimising the reaction conditions and reducing the solids loading to 2 wt%, the levulinic acid yield could be increased to >70%.

The hemicellulose sugars were concentrated (to a 70/30 GVL/water ratio) and used as feedstock to produce furfural. By increasing the temperature to 225°C in less than 30 second, a furfural yield >75 mol% of xylose was achieved for all feedstock using the sulfuric acid remaining in the solution from the fractionation step as catalyst.

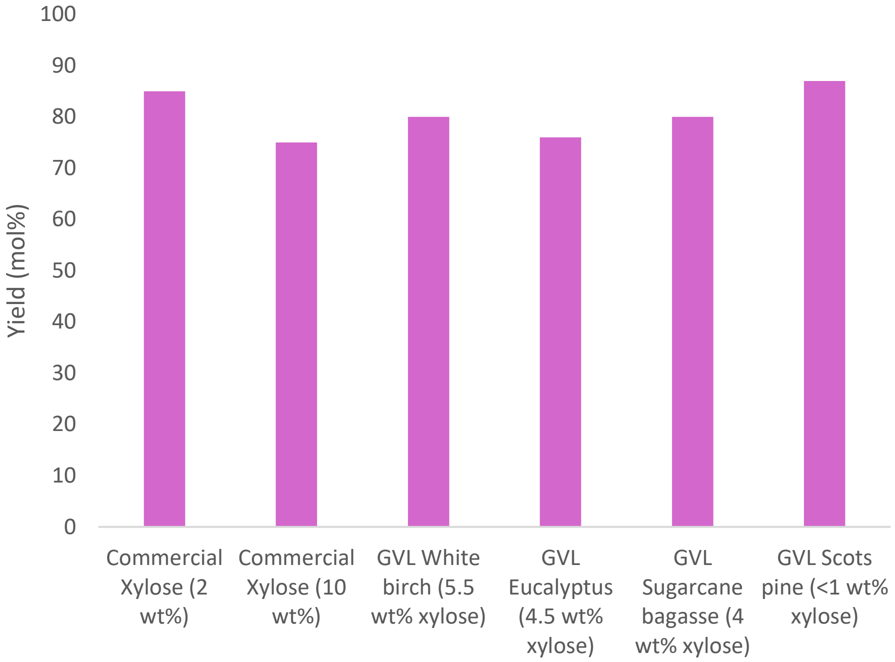

Figure S6. Maximum furfural yield achieved

6. Depolymerisation experiments

6.1. Sample work-up

After the reaction mixture containing depolymerised sample, residual lignin, catalyst, char, and solvent was collected from the reactor it was filtered. A sample for GC-MS analysis was taken from the filtrate. This was followed by the ethanol being evaporated from the filtrate with rotary evaporation producing the ethanol soluble lignin oil. The remaining solid consisting of residual lignin, catalyst and biochar was dissolved in THF to remove the residual lignin from the catalyst and char. The residual lignin dissolved in THF was filtrated and THF was evaporated using rotary evaporation. This resulted in the THF soluble portion containing residual lignin. The solid remaining after this consisted of catalyst and char. Figure S7Figure S7 displays the process flow for the sample treatment and highlights the three main fractions collected over the course of the experiments. Each fractions mass was recorded and compared to initial lignin mass loaded to the reactor to determine the weight percentage of the fractions. The bio-oil and residual lignin fractions were analysed using SEC. The bio-oil was analysed for product monomers using GC-MS and the char plus catalyst solid was not analysed.

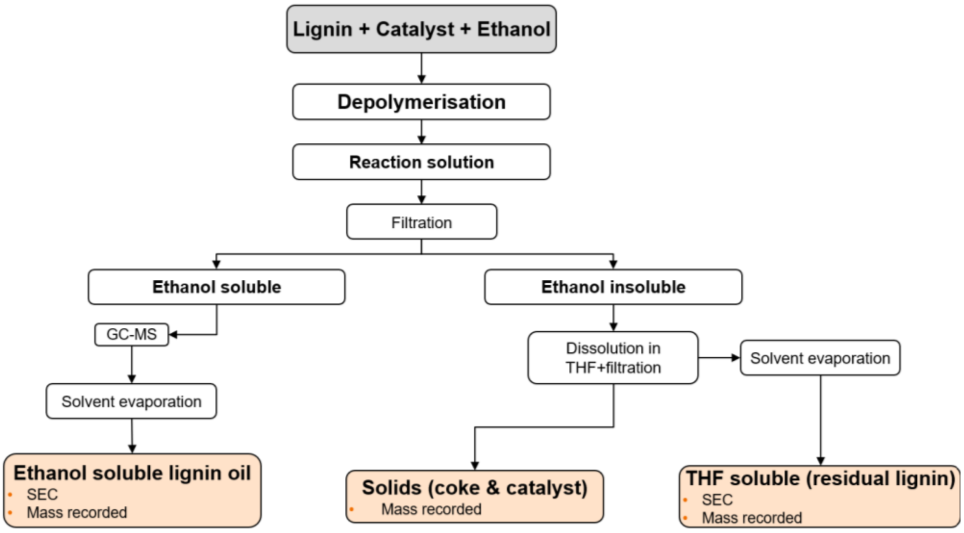

Figure S7. Process flow of sample treatment

6.2. Detailed results

Table S5 presents results from the depolymerisation experiments, excluding the DoE experiments. The table includes reaction conditions, mass balances of the produced fractions, monomer yields and molecular weights of different fractions. If no value is stated for the Mw of THF soluble portion, this indicates that sample was not soluble in the NaOH and could not be analysed. It is thought that the THF soluble portion has high molecular weight and condensed structures making it poorly soluble in NaOH. Additionally, the results from the DoE study are presented in Table S8.

The lignin monomers were identified and quantified by GC-MS from the ethanol solution after filtering the reaction mixture. H/G/S- ratios were calculated from monomers determined from lignin oils and were compared to the H/G/S-ratio of the original EtOH eucalyptus lignin. The EtOH eucalyptus depolymerisation experiments GC-MS results are presented below.

HSQC NMR was performed on the EtOH eucalyptus lignin and one of the lignin oils formed from its depolymerisation at 280 °C with 20 bar H_2 loading and 5 wt% MoC-1 catalyst (Figure S8).

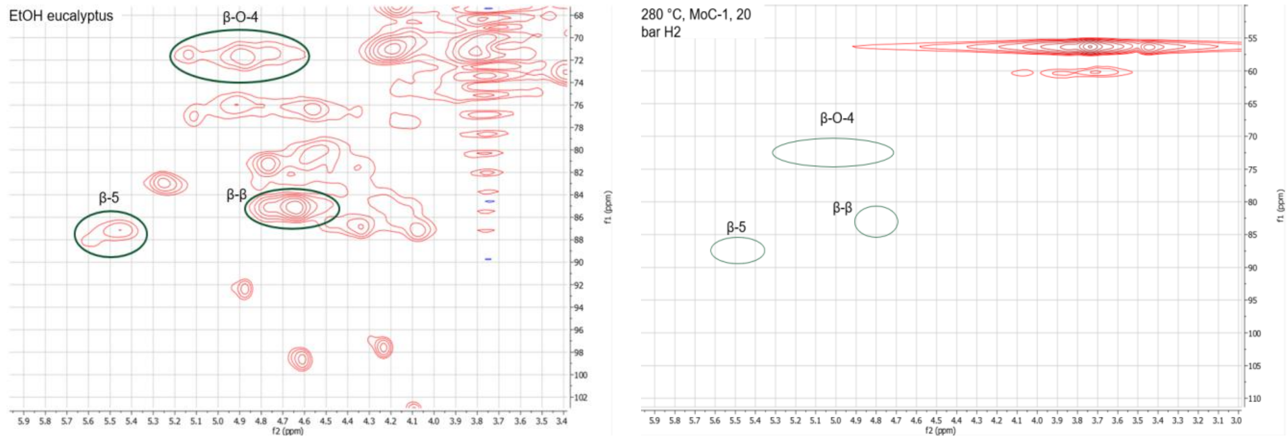

Figure S8. HSQC NMR spectra of EtOH eucalyptus lignin and the depolymerised lignin oil formed under conditions of 280 °C, 20 bar H2, 5 wt% MoC-1 catalyst.

Table S5. Reaction conditions, yields of lignin oil, residual lignin, biochar and monoaromatics. Molecular weights of EtOH and THF soluble fractions.

Entry	Lignin	Lignin (g)	EtOH (mL)	Catalyst (wt%)	H_2 (bar)	Temp. (°C)	Lignin oil wt%	Residual lignin wt%	Bio-char wt%	Total mass balance (wt%)	Monomers (wt% of lignin)	Mw EtOH soluble (Daltons)	Mw THF soluble (Daltons)[a]	Reactor
1	EtOH eucalyptus	2	80	-	-	280	47.1	2.9	47.6	97.6	18.8	1532	3856	200ml
2	EtOH eucalyptus	2	80	MoC-2 (5wt%)	-	280	64.5	14.0	18.0	96.5	14.3	1747	3696	200ml
3	EtOH eucalyptus	2	80	-	20	280	68.8	10.7	23.8	103.3	21.4	1893	6974	200ml
4	EtOH eucalyptus	2	80	MoC-3 (5 wt%)	-	280	51.0	13.0	43.0	107.0	22.8	1588	4785	200ml
5	EtOH eucalyptus	2	80	MoC-3 (5 wt%)	20	280	59.9	17.3	8.9	86.1	14.4	1907	6845	200ml
6	EtOH eucalyptus	2	80	MoC-2 (5 wt%)	20	280	88.6	11.4	1.5	101.5	14.1	1932	-	200ml
7	EtOH eucalyptus	2	80	MoP-1 (5 wt%)	-	280	46.6	10.7	36.9	94.2	14.5	1628	3969	200ml
8	EtOH eucalyptus	2	80	MoP-1 (5 wt%)	20	280	59.0	29.5	6.0	94.5	11.9	2166	5831	200ml
9	EtOH eucalyptus	2	80	MoP-2 (5wt%)	-	280	47.0	5.4	39.6	92.1	13.8	2132	5160	200ml
10	EtOH eucalyptus	2	80	MoP-2 (5wt%)	20	280	76.6	15.5	6.6	98.7	13.3	2115	6752	200ml
11	GVL eucalyptus	2	80	MoP-2 (5wt%)	20	280	63.9	18.3	6.9	89.1	15.1	2238	-	200ml
12	EtOH eucalyptus	2	80	MoP-2 (5wt%)	20	315	44.0	4.6	34.8	83.4	10.5	816	-	200ml
13	EtOH eucalyptus	4	160	MoP-2 (5wt%)	20	315	78.7	19.7	3.0	101.4	14.0	1293	2766	1L
14	GVL eucalyptus	4	160	MoP-2 (5wt%)	20	315	74.1	9.6	3.3	87.0	16.3	1272	3519	1 L
15	GVL sugarcane bagasse 1	4	160	MoP-2 (5wt%)	20	315	76.8	6.5	1.4	84.7	15.5	1227	3272	1 L
16	GVL white birch 1	4	160	MoP-2 (5wt%)	20	315	64.8	21.7	1.7	88.2	13.2	1065	-	1 L
17	GVL Scots pine	4	160	MoP-2 (5wt%)	20	315	86.1	12.7	2.0	100.7	7.0	717	-	1 L
18	EtOH eucalyptus	4	160	-	20	315	43.6	4.7	52.1	100.4	13.3	1174	3258	1 L
19	EtOH eucalyptus	2	80	MoC-3 (5 wt%)	-	280	48.3	13.1	51.8	113.2	15.4	1053	2715	200ml
20	EtOH eucalyptus	2	80	MoC-3 (5 wt%)	20	280	75.9	14.6	9.7	100.2	11.0	1266	4301	200ml
21	EtOH eucalyptus	2	80	MoC-1 (5wt%)	-	280	48.5	12.7	35.6	96.9	12.6	1188	3522	200ml
22	EtOH eucalyptus	2	80	MoC-1 (5wt%)	20	280	70.0	10.0	5.0	85.0	9.3	1315	4638	200ml
23	GVL sugarcane bagasse 2	2	80	MoC-1 (20wt%)	40	350	92.8	2.3	0	95.1	11.6	976*	-	200ml
24	GVL sugarcane bagasse 2	2	80	MoC-1 (20wt%)	40	280	75.9	4.1	0	80.0	11.4	1337*	-	200ml
25	GVL white birch 2	4	80	MoC-1 (10wt%)	20	315	84.7	1.2	0	85.9	13.3	1143*	-	200ml
26	GVL white birch 2	4	80	MoC-1 (10wt%)	40	315	86.2	0.4	0	86.6	13.1	1160*	-	200ml

* New SEC column. [a] In case the value is missing, the sample was insoluble in NaOH for the SEC analysis.

6.3. Monomer analysis

Table S6. Monoaromatics (wt%) of depolymerisation of EtOH eucalyptus lignin in ethanol (2 g lignin, 5 wt% catalyst, 80 ml EtOH).

**Compound**	**Origin**	**Side chain structure**	**Thermal**	**Thermal, 20 bar H2**	**MoC-1**	**MoC-1, 20 bar H2**	**MoC-2**	**MoC-2, 20 bar H2**	**MoC-3**	**MoC-3, 20 bar H2**	**MoP-1**	**MoP-1, 20 bar H2**	**MoP-2**	**MoP-2, 20 bar H2**	**MoP-2, 20 bar H2, 315 C**
**Entry in table S5**			1	3	21	22	2	6	4	5	7	8	9	10	12
Phenol	H	-H	0.05	0.01	0.01	0.00	0.01	0.03	0.18	0.03	0.06	0.01	0.01	0.01	0.00
o-Cresol	H	-CH3	0.00	0.00	0.00	0.00	0.00	0.01	0.08	0.01	0.03	0.01	0.00	0.00	0.00
m-Cresol	H	-CH3	0.01	0.00	0.01	0.00	0.01	0.01	0.05	0.01	0.02	0.01	0.00	0.00	0.00
p-Cresol	H	-CH3	0.00	0.00	0.00	0.00	0.00	0.03	0.32	0.03	0.10	0.01	0.00	0.00	0.01
4-Ethylphenol	H	-CH2CH3	0.00	0.00	0.00	0.00	0.00	0.00	0.02	0.00	0.01	0.00	0.00	0.00	0.00
2-Propylphenol	H	-CH2CH2CH3	0.00	0.00	0.00	0.00	0.00	0.00	0.01	0.00	0.00	0.00	0.00	0.00	0.00
4-Propylphenol	H	-CH2CH2CH3	0.18	0.00	0.02	0.00	0.01	0.00	0.01	0.00	0.00	0.00	0.00	0.00	0.00
Guaiacol	G	-H	0.47	0.26	0.44	0.20	0.25	0.17	0.49	0.23	0.31	0.16	0.27	0.16	0.22
4-Methylguaiacol	G	-CH3	0.31	0.28	0.28	0.16	0.20	0.15	0.36	0.20	0.28	0.17	0.24	0.18	0.22
4-Ethylguaiacol	G	-CH2CH3	0.13	0.14	0.13	0.10	0.11	0.10	0.17	0.11	0.13	0.09	0.11	0.10	0.08
4-Propylguaiacol	G	-CH2CH2CH3	1.84	1.94	0.52	0.27	0.43	0.41	0.63	0.34	0.43	0.34	0.32	0.33	0.47
Eugenol	G	-CH2CH=CH2	0.01	0.02	0.01	0.00	0.01	0.01	0.01	0.01	0.01	0.01	0.00	0.01	0.00
trans-Isoeugenol	G	-CH=CHCH3	0.50	1.11	0.13	0.14	0.00	0.00	0.15	0.14	0.00	0.07	0.00	0.08	0.00
Vanillin	G	-CHO	0.06	0.06	0.08	0.11	0.04	0.05	0.04	0.04	0.03	0.04	0.03	0.05	0.00
Acetoguaiacone	G	-COCH3	0.37	0.53	0.32	0.28	0.37	0.36	0.53	0.38	0.34	0.28	0.30	0.36	0.14
Guaiacylacetone	G	-CHCOCH3	1.21	0.85	0.34	0.30	0.53	0.45	0.57	0.38	0.44	0.35	0.40	0.43	0.16
Vanillic acid ethyl ester	G	-COOC2CH3	0.55	0.49	0.46	0.32	0.53	0.41	0.63	0.37	0.52	0.37	0.52	0.42	0.39
Syringol	S	-CH3	1.89	1.10	1.49	0.71	0.84	0.59	2.26	0.95	0.87	0.55	0.99	0.58	0.66
4-Methylsyringol	S	-CH2CH3	1.75	1.79	1.66	1.00	1.23	1.03	2.44	1.31	1.61	1.13	1.61	1.24	1.31
4-Ethylsyringol	S	-CH2CH2CH3	2.37	3.27	1.93	1.52	2.45	2.07	3.57	2.42	2.44	2.03	2.30	2.28	2.14
4-Propylsyringol	S	-CH2CH2CH3	1.65	2.33	1.47	1.40	1.74	2.06	2.70	2.04	2.03	1.98	1.84	2.01	2.08
4-Allylsyringol	S	-CH2CH=CH2	0.20	0.12	0.11	0.22	0.08	0.13	0.08	0.07	0.00	0.00	0.09	0.08	0.12
4-Propenylsyringol	S	-CH=CHCH3	0.45	0.51	0.36	0.43	0.79	1.21	0.53	0.47	0.56	0.66	0.55	0.74	0.57
α-oxyprpiosyringone	S	-COCOCH3	1.13	1.32	0.89	0.68	1.18	0.93	1.81	0.99	1.14	0.83	1.19	0.83	0.51
Acetosyringone	S	-COCH3	1.09	1.85	0.00	0.00	1.21	1.22	1.83	1.28	0.92	0.87	1.01	1.16	0.40
Syringylacetone	S	-CHCOCH3	1.59	2.18	1.11	0.99	1.29	1.71	2.04	1.51	1.31	1.15	1.21	1.39	0.43
Propiosyringone	S	-COCH2CH3	0.97	1.22	0.83	0.49	0.99	0.98	1.32	1.04	0.88	0.78	0.82	0.87	0.53
Monomers total			18.78	21.36	12.60	9.34	14.28	14.10	22.82	14.38	14.49	11.89	13.84	13.32	10.46
**Selectivity (wt%)**															
Saturated			56.8	52.0	63.2	57.6	50.9	47.2	58.2	53.5	57.5	54.6	55.7	51.8	68.9
Unsaturated			6.2	8.2	4.8	8.5	6.1	9.5	3.4	4.8	3.9	6.2	4.7	6.9	6.7
Oxygenated			37.1	39.8	32.0	33.9	43.0	43.3	38.5	41.7	38.6	39.2	39.6	41.3	24.4
H%			1.3	0.0	0.3	0.1	0.2	0.6	2.9	0.5	1.5	0.3	0.1	0.1	0.3
G%			29.0	26.6	21.5	20.2	17.3	15.0	15.7	15.3	17.2	15.8	15.8	15.9	16.1
S%			69.7	73.5	78.2	79.7	82.6	84.6	81.4	84.0	81.2	84.0	83.9	83.9	83.6

The GC-MS results from Table S6 have also been grouped into the graphs showing H/G/S selectivity, and side chain structure (Figure S9).

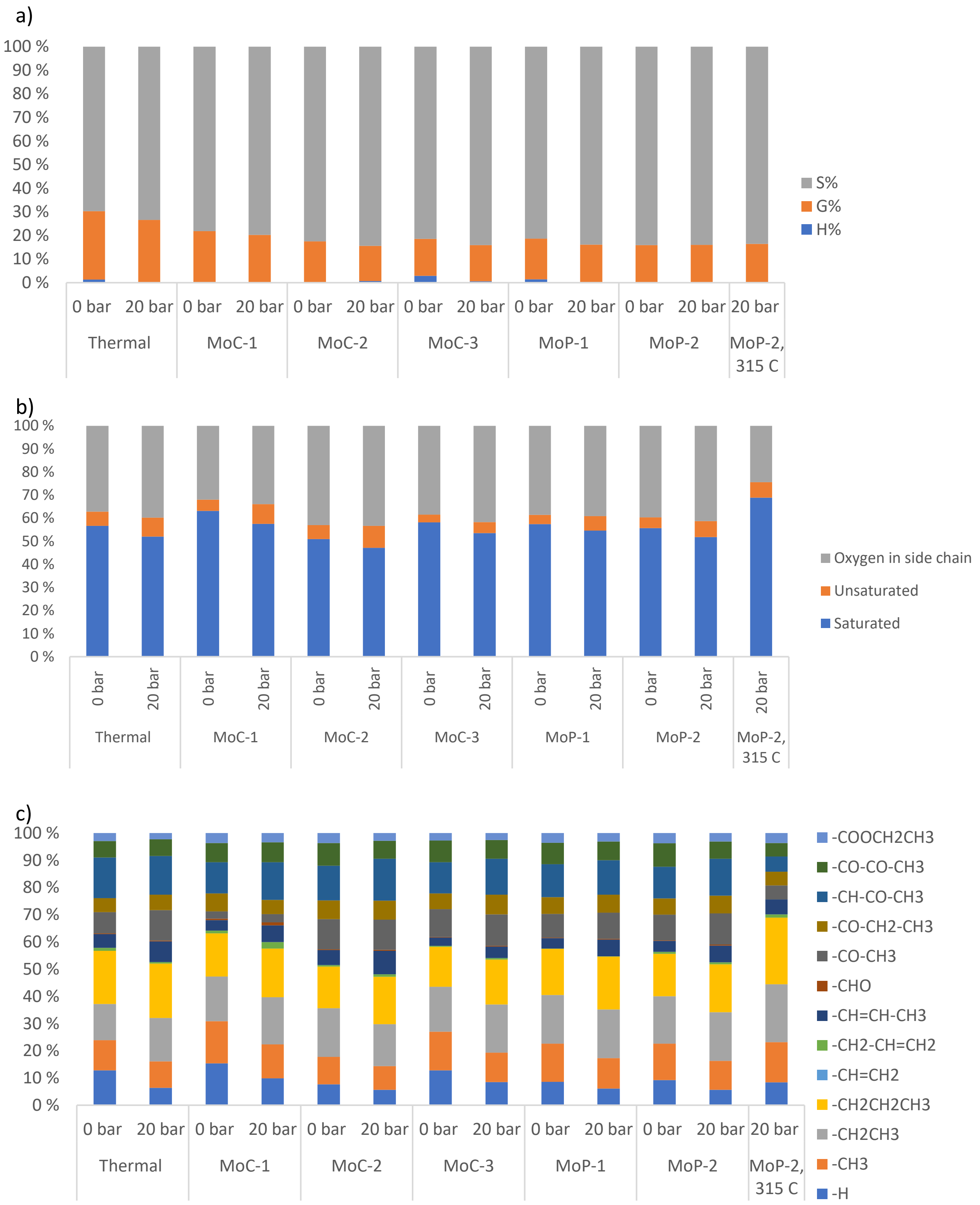

Figure S9. GC-MS results of monomers in the catalyst screening experiments Reaction conditions: 2 g EtOH eucalyptus lignin, 80 ml EtOH, 280 °C, 5 wt% catalyst. a) H/G/S ratio of monomers. b) Side chain groups on monomers, saturated, unsaturated and oxygen in the side chain. c) Type of side chain on monomers.

Table S7. Monoaromatics (wt%) of depolymerised GVL lignin.

Compound	Origin	Side chain structure	EtOH Eucalyptus	GVL Eucalyptus	GVL sugarcane bagasse 1	GVL white birch 1	GVL Scots pine
**Entry in table S3**			13	14	15	16	17
Phenol	H	-H	0.016	0.01	0.25	0.02	0.026
o-Cresol	H	-CH3	0.000	0.00	0.02	0.00	0.006
m-Cresol	H	-CH3	0.016	0.01	0.17	0.01	0.020
p-Cresol	H	-CH3	0.031	0.01	0.01	0.02	0.032
4-Ethylphenol	H	-CH2CH3	0.000	0.00	1.90	0.00	0.000
2-Propylphenol	H	-CH2CH2CH3	0.000	0.00	0.00	0.00	0.000
4-Propylphenol	H	-CH2CH2CH3	0.000	0.00	0.04	0.00	0.018
Guaiacol	G	-H	0.315	0.25	0.26	0.21	0.447
4-Methylguaiacol	G	-CH3	0.299	0.28	0.35	0.31	0.720
4-Ethylguaiacol	G	-CH2CH3	0.142	0.18	0.47	0.15	0.345
4-Propylguaiacol	G	-CH2CH2CH3	2.139	1.71	1.91	1.24	3.431
Eugenol	G	-CH2CH=CH2	0.000	0.00	0.00	0.01	0.024
trans-Isoeugenol	G	-CH=CHCH3	0.000	0.00	0.13	0.00	0.117
Vanillin	G	-CHO	0.000	0.00	0.02	0.11	0.000
Acetoguaiacone	G	-COCH3	0.173	0.22	0.32	0.27	0.389
Guaiacylacetone	G	-CHCOCH3	0.362	0.47	0.38	0.35	0.601
Vanillic acid ethyl ester	G	-COOCH2CH3	0.503	0.40	0.58	0.39	0.632
Syringol	S	-CH3	0.834	0.79	0.40	0.49	0.011
4-Methylsyringol	S	-CH2CH3	1.494	1.54	0.85	1.24	0.025
4-Ethylsyringol	S	-CH2CH2CH3	2.454	2.64	1.84	1.78	0.144
4-Propylsyringol	S	-CH2CH2CH3	2.690	3.69	3.99	3.57	0.000
4-Allylsyringol	S	-CH2CH=CH2	0.126	0.13	0.17	0.03	0.000
4-Propenylsyringol	S	-CH=CHCH3	0.818	0.92	0.30	0.58	0.000
α-oxyprpiosyringone	S	-COCOCH3	0.629	0.58	0.32	0.42	0.000
Acetosyringone	S	-COCH3	0.378	0.53	0.22	0.43	0.000
Syringylacetone	S	-CHCOCH3	0.315	1.23	0.37	0.95	0.000
Propiosyringone	S	-COCH2CH3	0.291	0.71	0.25	0.60	0.000
Monomers total			14.0	16.33	15.53	13.20	6.99
**Selectivity (wt%)**							
Saturated %			74.4	68.1	80.3	68.5	74.8
Unsaturated %			6.7	6.4	3.8	4.8	2.0
Oxygenated %			18.9	25.4	15.9	26.7	23.2
H%			0.4	0.3	15.4	0.4	1.4
G%			28.0	21.5	28.5	23.1	96.0
S%			71.5	78.2	56.1	76.5	2.6

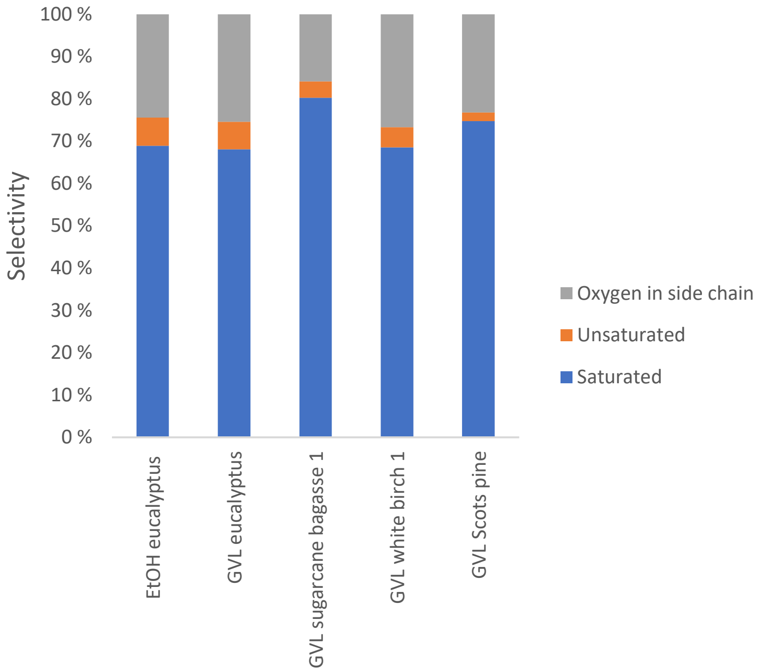

Figure S10. GC-MS results of monomers in different feedstock experiments. Reaction conditions 315 °C, 5 wt% MoP-2 catalyst, 20 bar H2. Side chain groups on monomers, saturated, unsaturated and oxygen in the side chain.

6.4. Design of experiments study

The experimental matrix and the results of the DoE study are shown in Table S8. The results of the depolymerisation experiments were fitted using MODDE software version 13.0.2 using multiple linear regression (MLR). A good fit was achieved for lignin-oil yield (R2=0.83, F=19, p=0.001), char yield (R2=0.69, F=5, p=0.038), reduction of Mw (R2=0.85, F=14, p=0.003), amount of aliphatic hydroxyls (R2=0.90, F=16, p=0.004) and selectivity to deoxygenated side chains (R2=0.75, F=7, p=0.016), while monomer yield (R2=0.54, F=3, p=0.122) showed moderate fit. The observed vs. predicted values are presented Figure S11 and the coefficients in Figure S12. The response surfaces are shown in Figure S13 and Figure S14.

Table S8. Experimental data for DoE experiments

Entry	MoC-1 Catalyst (wt%)	H_2 (bar)	Temp. (°C)	Lignin oil wt%	Residual lignin wt%	Bio-char wt%	Total mass balance (wt%)	Monomers (wt% of lignin)	Aliphatic -OH (mmol/g)	Selectivity to deoxyg. side chains (mol%)	Mw Reduction	Mw EtOH soluble (Daltons)
**1**	0	-	280	55.0	5.0	25.0	85.0	11.8	0.36	64.2	29.5	1980
**2**	20	-	280	78.5	12.3	7.7	98.5	10.1	0.22	71.6	46.1	1513
**3**	0	40	280	60.2	17.7	3.7	81.5	10.2	0.52	60.5	23.8	2139
**4**	20	40	280	88.2	1.6	0	89.8	8.9	0.7	82	47.0	1489
**5**	10	20	315	66.4	11.9	0	78.4	10.4	0.36	91.7	56.9	1210
**6**	10	20	315	73.6	2.3	0.7	76.6	11.2	0.37	88.2	55.6	1245
**7**	10	20	315	77.7	1.6	0	79.3	12.1	0.36	90.7	59.2	1145
**8**	0	-	350	35.3	0.5	43.2	79.0	10.0	0.54	80.8	54.6	1274
**9**	20	-	350	68.2	5.9	19.3	93.4	11.2	0.26	85.4	60.7	1103
**10**	0	40	350	52.2	1.2	30.2	83.7	8.6	0.84	97.3	53.3	1310
**11**	20	40	350	91.9	1.0	0	92.9	7.3	1	99.9	64.1	1008

All DoE experiments used 2 g GVL white birch lignin 2 and 80ml EtOH in 200 mL reactor.

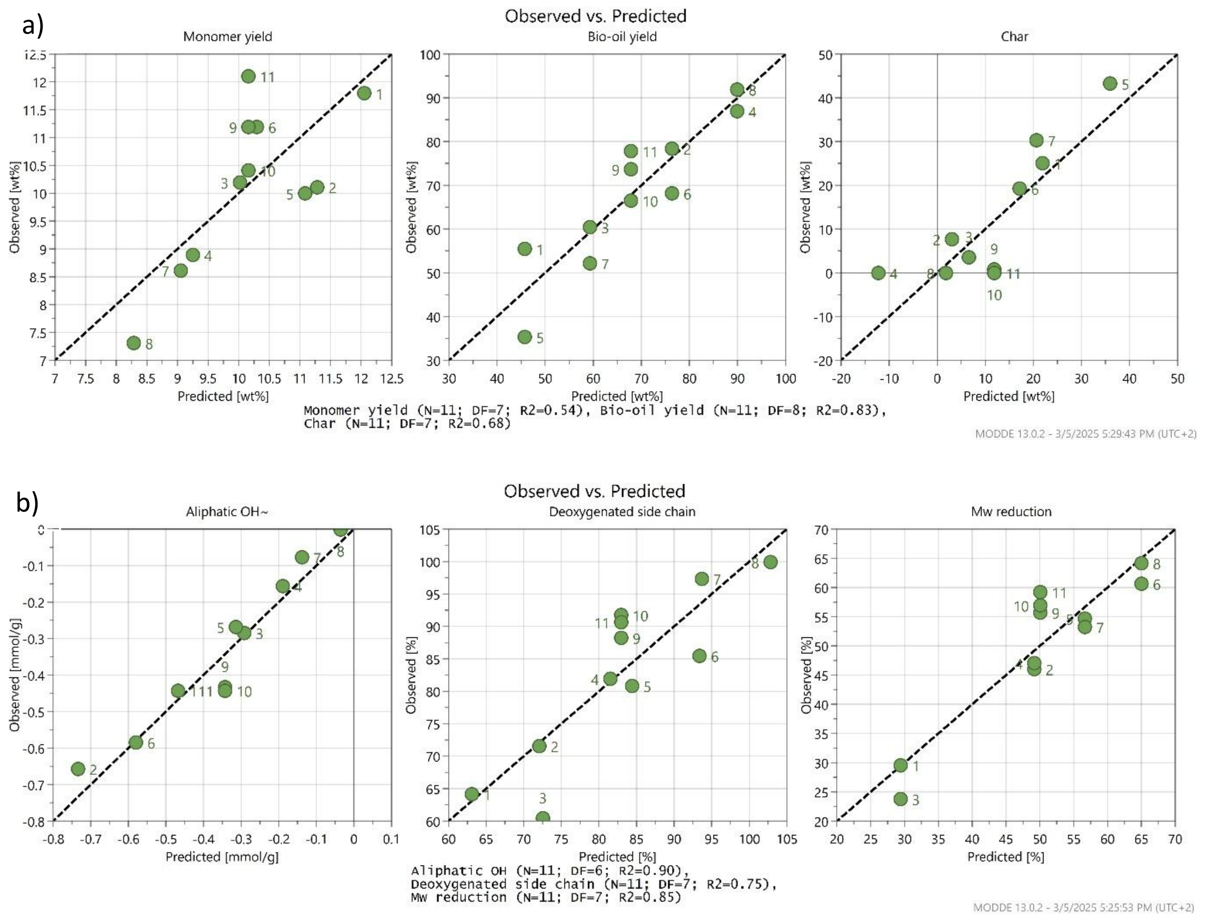

Figure S11. Observed vs. predicted values for a) yield of monomers, lignin oil and char and b) aliphatic hydroxyls, selectivity to deoxygenated side chains and reduction of Mw.

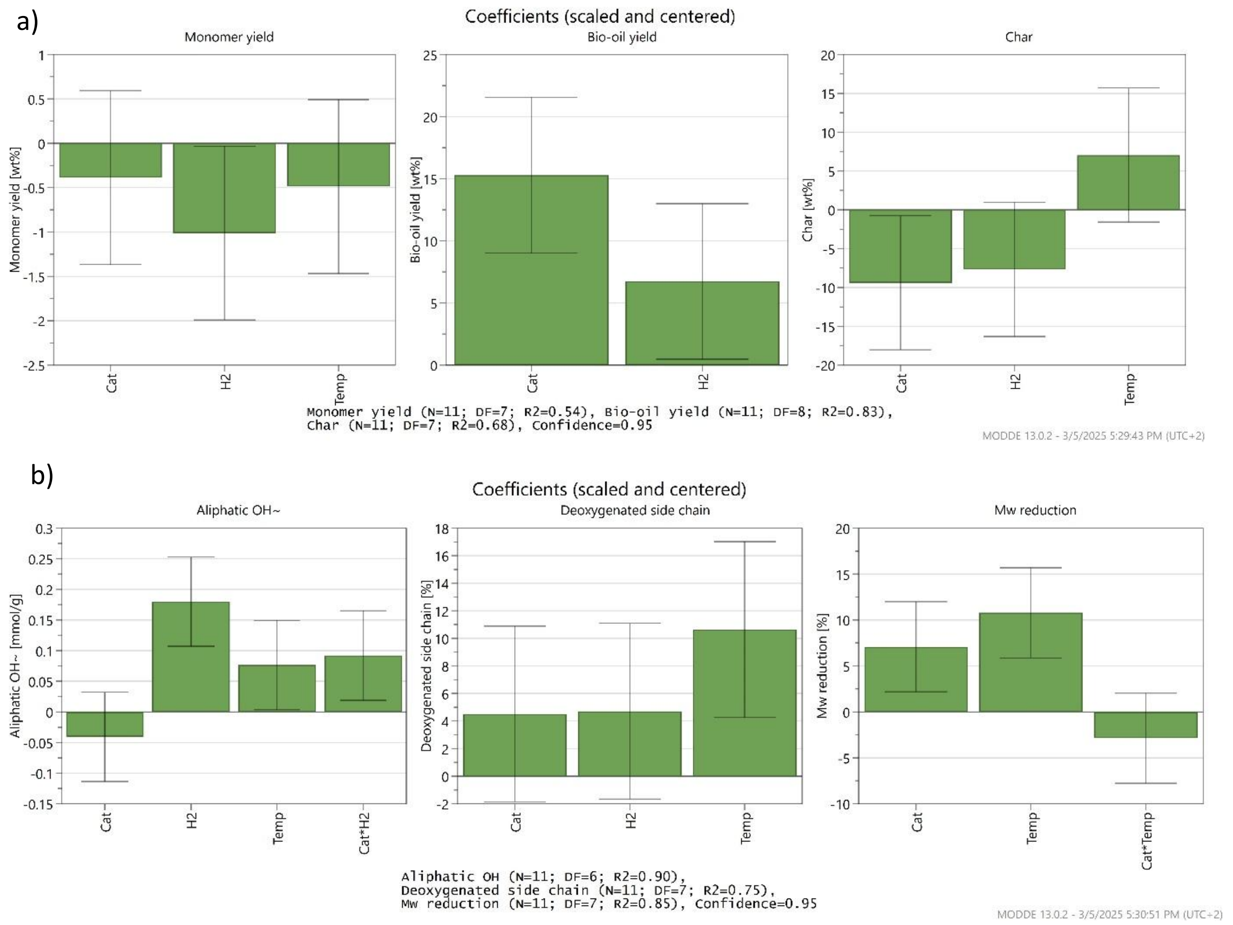

Figure S12. Coefficients for a) yield of monomers, lignin oil and char and b) aliphatic hydroxyls, selectivity to deoxygenated side chains and reduction of Mw.

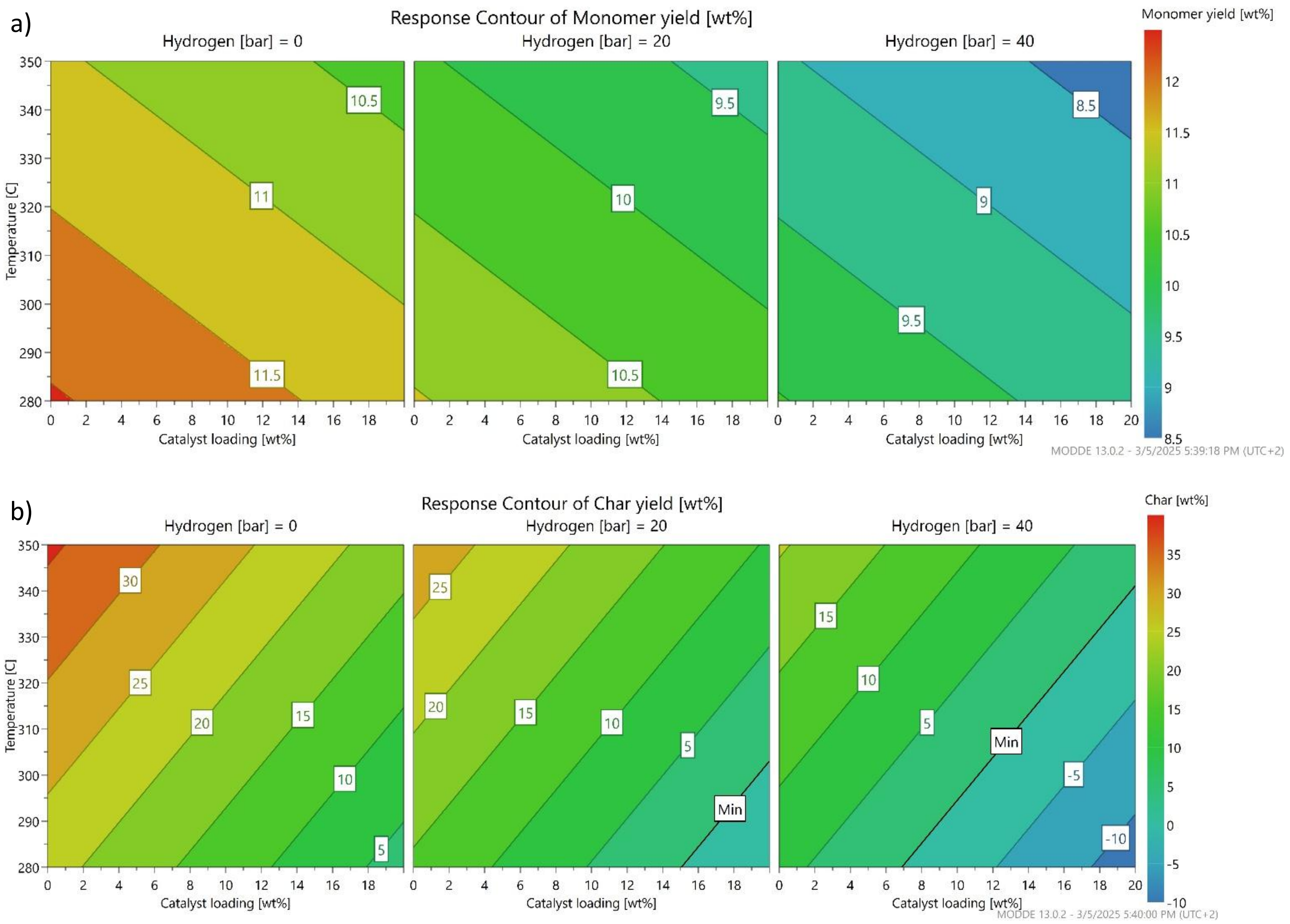

Figure S13. Response surfaces for the yield of a) monomers and b) char.

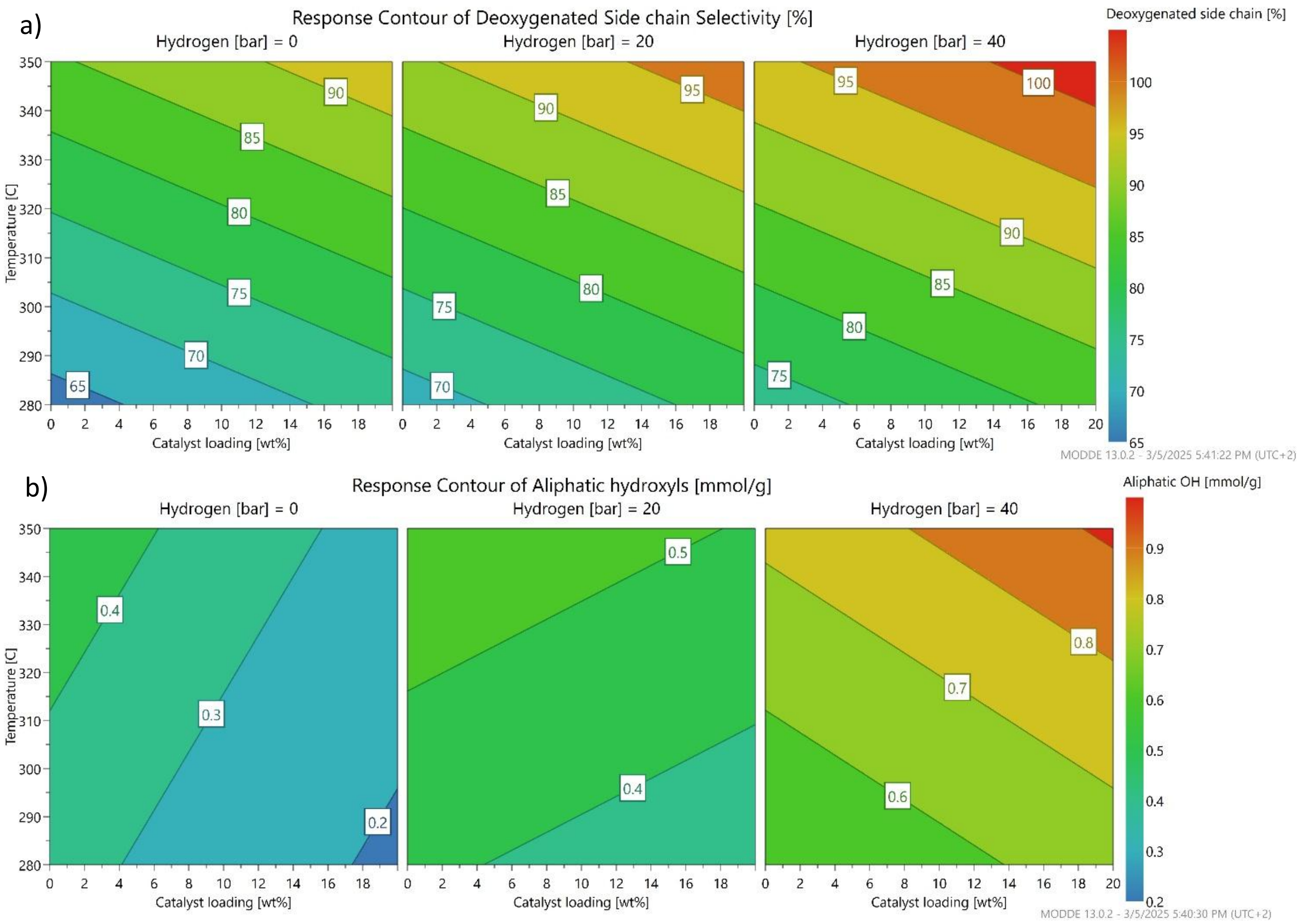

Figure S14. Response surfaces for a) selectivity to deoxygenated side chains and b) amount of aliphatic hydroxyls.

6.5. Analysis of hydroxyl groups by ^{31}P-NMR

Table S9. ^{31}P-NMR results for DoE experiments of white birch depolymerisation. Results are given as mmol/g.

**Sample**	**Aliphatic OH (150-145 ppm)**	**Carboxylic acid (136-134 ppm)**	**Condensed and Syringyl phenolic units (145-140.5ppm)**	**Guaiacyl (140-140.5 ppm)**	**Catechols (139.5-138.5 ppm)**	**p-OH-phenyl (138.5-137 ppm)**	**Total phenolic OH**	**Total OH**
GVL white birch 2	2.04±0.09	0.16±0.01	2.07±0.07	0.31±0.01	0.24±0.02	0.08±0.01	2.70±0.10	4.90±0.20
Thermal, 280 °C, no H2	0.36±0.03	0.10±0.02	2.88±0.01	0.69±0.03	0.55±0.02	0.31±0.02	4.43±0.08	4.89±0.13
Thermal, 280 °C, 40 bar H2	0.52±0.01	0.09±0.00	2.94±0.05	0.63±0.03	0.44±0.01	0.26±0.01	4.28±0.11	4.89±0.12
Thermal, 350 °C (310 °C), no H2	0.54±0.01	0.22±0.01	2.93±0.04	0.82±0.01	0.72±0.02	0.35±0.01	4.82±0.08	5.59±0.10
Thermal, 350 °C, 40 bar H2	0.84±0.01	0.30±0.01	3.13±0.14	0.60±0.00	1.31±0.04	0.74±0.05	5.77±0.23	6.92±0.25
10 wt% MoC-1, 315 °C, 20 bar H2	0.37±0.02	0.21±0.00	2.84±0.01	0.66±0.01	0.59±0.01	0.41±0.01	4.50±0.04	5.08±0.06
10 wt% MoC-1, 315 °C, 20 bar H2	0.37±0.01	0.19±0.01	2.86±0.16	0.72±0.03	0.62±0.03	0.47±0.02	4.67±0.25	5.23±0.26
10 wt% MoC-1, 315 °C, 20 bar H2	0.36±0.00	0.16±0.02	2.95±0.04	0.66±0.00	0.60±0.03	0.37±0.01	4.59±0.08	5.11±0.10
20 wt% MoC-1, 350 °C, 40 bar H2	1.00±0.09	0.22±0.02	1.98±0.07	0.37±0.01	0.69±0.02	0.57±0.03	3.61±0.12	4.83±0.24
20 wt% MoC-1, 350 °C, no H2	0.26±0.01	0.36±0.01	2.53±0.02	0.59±0.01	0.53±0.01	0.37±0.03	4.03±0.07	4.64±0.09
20 wt% MoC-1, 280 °C, 40 bar H2	0.70±0.01	0.05±0.00	2.85±0.06	0.74±0.01	0.46±0.01	0.24±0.00	4.28±0.08	5.03±0.09

6.6. Additional experiments

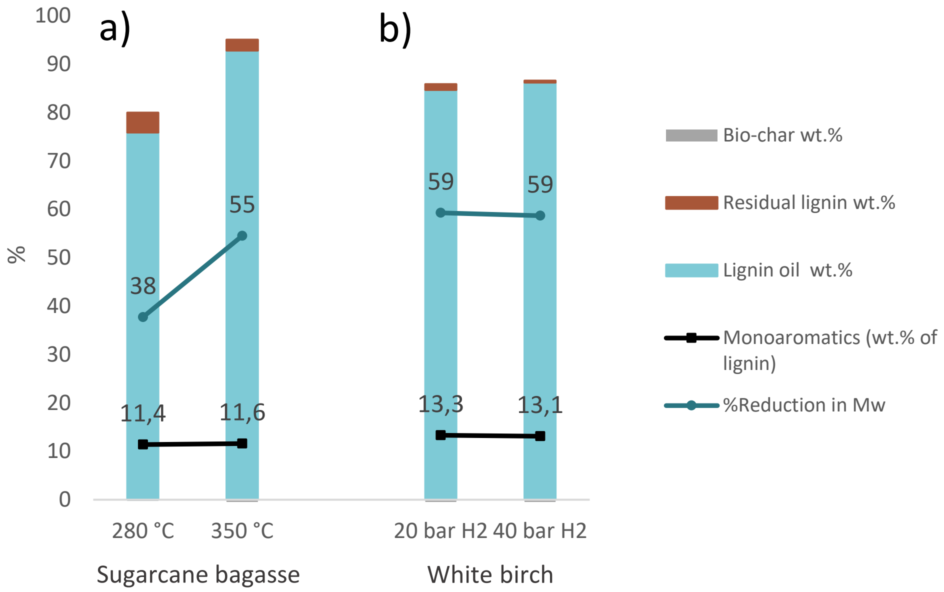

Figure S15. Depolymerisation of GVL lignins. a) Sugarcane bagasse depolymerisations using 2 g lignin, 80 ml EtOH, 20 wt% MoC-1 and 40 bar H_2. b) Depolymerisation of GVL white birch 2 with higher lignin concentration. Reaction conditions: 4 g lignin, 80 ml EtOH, 10 wt% MoC-1, 40 bar H_2.

7. References

[1] C. Sener, A. H. Motagamwala, D. M. Alonso, J. A. Dumesic, *ChemSusChem* **2018**, *11*, 2321–2331.

[2] T. Ohra-Aho, P. Niemi, A.-M. Aura, M. Orlandi, K. Poutanen, J. Buchert, T. Tamminen, *J Agric Food Chem* **2016**, *64*, 812–820.

[3] J. C. Del Río, J. Rencoret, P. Prinsen, Á. T. Martínez, J. Ralph, A. Gutiérrez, *J Agric Food Chem* **2012**, *60*, 5922–5935.

[4] M. BakerS, *Holzforschung* **1996**, *50*, 573–574.

[5] C. Tang, H. Zhang, K. Xu, Q. Zhang, J. Liu, C. He, L. Fan, T. Asefa, *J Mater Chem A Mater* **2019**, *7*, 18030–18038.

[6] R. Ma, Y. Zhou, Y. Chen, P. Li, Q. Liu, J. Wang, *Angewandte Chemie International Edition* **2015**, *54*, 14723–14727.

[7] C. Stinner, R. Prins, T. Weber, *J Catal* **2000**, *191*, 438–444.

[8] R. Cheng, Y. Shu, L. Li, M. Zheng, X. Wang, A. Wang, T. Zhang, *Appl Catal A Gen* **2007**, *316*, 160–168.

[9] A. S. Jääskeläinen, T. Liitiä, A. Mikkelson, T. Tamminen, *Ind Crops Prod* **2017**, *103*, 51–58.

[10] A. Granata, D. S. Argyropoulos, *J Agric Food Chem* **1995**, *43*, 1538–1544.
